# Nuclear isoform of RAPH1 interacts with FOXQ1 to promote aggressiveness and radioresistance in breast cancer

**DOI:** 10.1038/s41419-023-06331-9

**Published:** 2023-12-07

**Authors:** Qun Liu, Yu Cao, Xiaolin Wei, Huiting Dong, Mengyao Cui, Shu Guan, Bo Liu, Xu Wang, Peng Xing

**Affiliations:** 1 Department of Surgical Oncology, Breast Surgery, General Surgery, First Hospital of China Medical University, Shenyang, China; 2 Department of Cardiac Surgery, First Hospital of China Medical University, Shenyang, China

**Keywords:** Breast cancer, Oncogenes

## Abstract

Radioresistance limits the efficacy of radiotherapy against breast cancer, especially the most lethal subtype of breast cancer, triple-negative breast cancer (TNBC). Epithelial-to-mesenchymal transition (EMT) is closely related to tumor radioresistance. In this work, we attempted to identify the key EMT-related transcription factor(s) that can induce radioresistance in breast cancer cells. A set of 44 EMT transcription factors were analyzed in parental and radioresistant TNBC cell lines. The function of FOXQ1, a differentially expressed transcription factor, was determined in TNBC radioresistance. FOXQ1-interacting proteins were identified by co-immunoprecipitation and mass spectrometry. Compared with parental cells, FOXQ1 was significantly upregulated in radioresistant TNBC cells. Silencing of FOXQ1 increased the radiosensitiviy of radioresistant TNBC cells both in vitro and in vivo. FOXQ1 associated with a nuclear isoform of RAPH1 (named RAPH1-i3) in radioresistant TNBC cells. Overexpression of RAPH1-i3 enhanced TNBC cell proliferation and migration, and most interestingly, induced radioresistance in parental TNBC cells when co-expressed with FOXQ1. Similar findings were observed in estrogen receptor-positive breast cancer cell lines that had co-expression of RAPH1-i3 and FOXQ1. Mechanistically, co-expression of RAPH1-i3 and FOXQ1 activated STAT3 signaling and increased the expression of CCND1, MCL1, Bcl-XL, and MMP2. Depletion of RAPH1-i3 impaired the radioresistance of radioresistant TNBC cells. Additionally, RAPH1-i3 upregulation was associated with advanced tumor stage and reduced disease-free survival in TNBC patients. These results collectively show that RAPH1-i3 interacts with FOXQ1 to promote breast cancer progression and radioresistance. RAPH1-i3 and FOXQ1 represent therapeutic targets for the treatment of breast cancer including TNBC.

## Introduction

Breast cancer is a common malignancy affecting women. Triple-negative breast cancer (TNBC) is the most lethal subtype of breast cancer and characterized by the absence of estrogen receptor, progesterone receptor, and human epidermal growth factor receptor 2 [1]. Treatment options available for TNBC are limited. Radiotherapy has been shown to offer therapeutic benefits to TNBC patients [2, 3]. A meta-analysis of 17 randomized trials reveals that radiotherapy after breast-conserving surgery significantly reduces the 10-year recurrence rate and the 15-year cancer-related death rate in TNBC patients [3]. However, a recent phase III randomized trial demonstrates that post-mastectomy radiotherapy only provides a modest improvement in locoregional control in selected TNBC patients [4]. Tumor radioresistance is a major obstacle that blunts the efficacy of radiotherapy against TNBC [5]. Therefore, many efforts have been made to identify the key regulators of TNBC radioresistance [5, 6].

Epithelial-to-mesenchymal transition (EMT) is a reversible phenotype change commonly occurring during tumor progression [7]. EMT endows cancer cells with survival advantage [8, 9]. Fischer et al. [8] reported that EMT tumor cells can survive cyclophosphamide treatment through upregulation of chemoresistance-related genes, and inhibiting EMT reverses the chemoresistance. Wang et al. [9] reported that 4.1 N loss-mediated induction of EMT promotes anoikis resistance in epithelial ovarian cancer cells. microRNA-375-dependent reversal of EMT restores the sensitivity of tamoxifen-resistant breast cancer cells to tamoxifen [10]. Upon radiotherapy, many types of cancer cells can undergo EMT to augment resistance [11–13]. For example, ionizing irradiation-treated breast cancer cells have increased expression of CD146, which promotes EMT, DNA damage repair, and radioresistance [13]. Targeting EMT represents a promising strategy to improve therapeutic response in tumor cells.

EMT can be initiated by a number of transcription factors [14]. Some of them show the ability to drive therapeutic resistance in cancer cells [15, 16]. For example, the EMT-inducing transcription factor ZEB1 is stabilized and promotes homologous recombination-dependent DNA repair and resistance to radiation in breast cancer cells [15]. The EMT transcription factor SLUG mediates chemotherapy resistance in pancreatic cancer, and its depletion increases the sensitivity of pancreatic cancer cells to gemcitabine [16]. The forkhead box (FOX) transcription factor FOXQ1 plays an important role in EMT and chemoresistance of breast cancer cells [17, 18]. Overexpression of FOXQ1 promotes EMT and resistance to chemotherapy-induced apoptosis in human mammary epithelial cells [17]. These studies encourage us to hypothesize that EMT-inducing transcription factors might contribute to the development of radioresistance in breast cancer.

Hence, in this work, we sought to identify the key EMT-related transcription factor(s) that renders breast cancer cells resistant to radiotherapy. As a result, FOXQ1 was found to be aberrantly upregulated in radioresistant TNBC cell lines and involved in TNBC radioresistance. The interaction between FOXQ1 and a nuclear isoform of RAPH1 enhanced progression and radioresistance in TNBC and estrogen receptor-positive breast cancer.

## Materials and methods

### Cell culture

Human TNBC cell lines (MDA-MB-231, MDA-MB-436, and MDA-MB-468) and estrogen receptor-positive breast cancer cell lines (MCF7 and T47D) were cultured in Dulbecco’s Modified Eagle Medium (DMEM) supplemented with 10% fetal bovine serum (FBS) and 100 U/mL penicillin-streptomycin (Sigma-Aldrich, St. Louis, MO, USA). All the cell lines were validated to have no mycoplasma contamination before use.

### Generation of radioresistant TNBC cell lines

The radioresistant TNBC cell lines MDA-MB-231-RR and MDA-MB-468-RR were generated by continuous radiation using a cabinet X-ray system, as described previously [19]. Briefly, parental TNBC cells were daily irradiated at a fractionated dose of 2 Gy (5 fractions per week) for 5 weeks, with a total dose of 50 Gy. The surviving clones were expanded and defined as radioresistant cells.

### Quantitative real-time PCR (qRT-PCR) analysis

Total RNA was extracted using TRIzol (Thermo Fisher Scientific, Carlsbad, CA, USA) according to the manufacturer’s protocol. Reverse transcription was conducted using the PrimeScript RT Reagent Kit (TaKaRa, Japan). qRT-PCR was performed using the iTaq Universal SYBR Green One-Step Kit (Bio-Rad, Hercules, CA, USA). GAPDH was used as the normalization control. Primer sequences for *TWIST1*, *KLF8*, *FOXM1*, *FOXQ1*, *SOX2*, *NOTCH1*, *CTNNB1*, *RAPH1*, *RAPH1-v1*, *RAPH1-v3*, *CCND1*, *MCL1*, *Bcl-XL*, and *MMP2* are listed in Supplementary Table S1, while those for the other genes tested are available on request. The results were analyzed according to the 2^−ΔΔCT^ method [20].

### Western blot analysis and cellular fractionation

Cells were lysed in ice-cold radioimmunoprecipitation assay (RIPA) buffer supplemented with a protease inhibitor cocktail (Cell Signaling Technology, Beverly, MA, USA). Protein concentrations were determined using a BCA protein assay kit (Beyotime, Beijing, China). Protein samples were resolved in sodium dodecyl sulfate–polyacrylamide gel electrophoresis (SDS-PAGE) and transferred to polyvinylidene difluoride membranes. The membranes were blocked in 5% fat-free milk and incubated with primary antibodies at 4 °C overnight. The membranes were then incubated with secondary antibodies and visualized using the ECL Plus Chemiluminescence Detection Kit (Thermo Fisher Scientific). The following antibodies were used as the primary antibodies: anti-FOXQ1 (Thermo Fisher Scientific), anti-RAPH1 (Thermo Fisher Scientific), anti-phospho-STAT3 (Cell Signaling Technology), anti-STAT3 (Cell Signaling Technology), anti-Myc-tag (Cell Signaling Technology), anti-CCND1 (Cell Signaling Technology), anti-MCL1 (Cell Signaling Technology), anti-Bcl-XL (Cell Signaling Technology), anti-MMP2 (Cell Signaling Technology), anti-β-tubulin (Beyotime), and anti-Lamin-B1 (Beyotime). In some experiments, cellular fractionation was performed using the Nuclear and Cytoplasmic Protein Extration Kit (Beyotime) according to the manufacturer’s instructions. The protein samples were subjected to Western blotting as described above.

### Immunofluorescence staining

Cells were seeded on glass coverslips in 24-well plates and cultured overnight. The cells were fixed using 4% paraformaldehyde, permeabilizated, and blocked with 0.5% bovine serum albumin (Beyotime). The cells were incubated with anti-FOXQ1 or anti-Myc tag antibody (Cell Signaling Technology) at 4 °C overnight, followed by incubation with secondary antibodies conjugated with Alexa Fluor 568 or Alexa Fluor 680 (Thermo Fisher Scientific). Nuclei were counterstained with 4’,6-diamidino-2-phenylindole (DAPI; Beyotime). Stained cells were imaged using a fluorescence microscope.

### Small interfering RNAs (siRNAs), plasmids, and transfections

To knockdown total RAPH1 mRNA levels, siRNA duplexes were designed to target both exons 7 and 9 that are included in all the *RAPH1* transcript variants. To selectively deplete the RAPH1-v1 (NM_213589) and -v3 (NM_203365) transcript variants, the siRNAs were designed to target the sequences unique to the 2 transcripts. The siRNA sense sequences are listed as follows: siRAPH1#1, 5′-AAUUAAAGAGGCACAAGUGTT-3′; siRAPH1#2, 5′-AAGCUUAUAUUUAUGGAGCTT-3′; siRAPH1-v3#1, 5′-CCAGCAAGUAAAACAAAUGTT-3′; siRAPH1-v3#2, 5′-GAUGUUCCAGCAAGCAGGCTT-3′; siRAPH1-v1#1, 5′-CCAAUUACUGAGGAAGAACTT-3′; siRAPH1-v1#2, 5′-AAGUUCAGCAUAUUACTCATT-3′. Non-targeting siRNA was used as a control (siCtrl). The coding sequence of *FOXQ1* (NM_033260) was cloned into the pcDNA3.1(-) vector. For expression of Myc-tagged RAPH1 isoforms, full-length RAPH1-v3 was amplified by standard PCR and cloned into the pcDNA3.1 Myc-His (-) A vector.

Transfection of the plasmids was performed using Lipofectamine 3000 transfection reagent (Thermo Fisher Scientific). The siRNAs were transfected using Oligofectamine (Thermo Fisher Scientific) at a concentration of 40-60 nM. In some experiments, MDA-MB-231 and MDA-MB-468 cells transfected with indicated constructs were treated with Stattic (2 μM, 6 h; Sigma-Aldrich), a potent STAT3 phosphorylation inhibitor [21].

### Generation of stably transfected cell lines

Short hairpin RNAs (shRNAs) targeting 2 different sites of *FOXQ1* and *RAPH1-v3* were cloned into the pLKO.1-puro vector (Addgene, Watertown, MA, USA). The shRNA targeting sequences were shown below: shFOXQ1#1, 5′-GGCTCTTTAACGAGCAGCAGA-3′, shFOXQ1#2, 5′-GAAAAGTGACTATCTCGGAAG-3′, shRAPH1-v3#1, 5′-GAAGAGTCCAGCAAGTAAAAC-3′, and shRAPH1-v3#2, 5′-TATTGACTTCACAGAAGAACA-3′. The sequence of a negative control shRNA (shCtrl) was 5′-CAACAAGATGAAGAGCACCAA-3′. The indicated shRNAs were transfected into radioresistant TNBC cells with Lipofectamine 3000. Four-eight hours later, the transfected cells were selected for stable clones using 2 μg/mL puromycin (Thermo Fisher Scientific).

### Cell viability assay

Cells were seeded into 96-well plates (3000 cells per well) and serum starved for 24 h before exposure to different doses of irradiation. Two days after irradiation, cell viability was determined using AlamarBlue (Thermo Fisher Scientific). Absorbance was measured at 570 nm. Percentage survival was calculated relative to non-irradiated control cells.

### Clonogenic assay

Cells were seeded into 6-well plates (200–300 cells per well) and exposed to different doses of irradiation. After culturing for 14 days, the cells were fixed, stained with 0.5% crystal violet (Sigma-Aldrich), and counted. Survival fraction was calculated as: (number of colonies/number of cells plated)_irradiated_/(number of colonies/number of cells plated)_non-irradiated_.

### Apoptosis analysis

Cell apoptosis was quantified by flow cytometry using the Annexin V-FITC/PI Apoptosis Detection Kit (Sigma‑Aldrich). In brief, cells were exposed to irradiation at 6 Gy for 48 h and harvested. The cells were resuspended in binding buffer containing fluorescein isothiocyanate (FITC)-labeled annexin-V and propidium iodide (PI) and incubated for 20 min in the dark. Cell apoptosis were analyzed by flow cytometry.

### Cell proliferation assay

Cells were seeded into 12-well plates (4 × 10^4^ cells per well) and cultured for 2-5 days. Cell number was determined at different time points. Accordingly, the cell proliferation curve was plotted.

### Transwell migration assay

Cells suspended in serum-free medium were seeded into the upper chambers of Transwell inserts (5 × 10^4^ cells per well). The medium containing 10% FBS was added to the lower chamber. After culturing for 16 h, the nonmigrating cells on the upper surface of the filter were removed. The cells on the lower surface of the membrane were stained with crystal violet and counted under a microscope.

### Tumor xenograft experiments

Radioresistant MDA-MB-231 cells transfected with FOXQ1 shRNA, RAPH1-v3 shRNA or both shRNAs were injected into mammary fat pads of female BALB/c nude mice (4 week old), 5 × 10^6^ cells per mouse. Five mice were randomly assigned to each group. At 1 week after cell injection, tumor-bearing mice received irradiation treatment or not. Irradiation was daily delivered to the xenograft tumors at a dose of 2 Gy for 5 continuous days [22]. Tumor volume was calculated every week. Mice were euthanized 5 weeks post-implantation, and xenograft tumors were removed and photographed. The animal experiment procedures were approved by the Ethics Committee of China Medical University (Shenyang, China).

### Co‐immunoprecipitation and mass spectrometry analysis

Radioresistant MDA-MB-231-RR cells (1 × 10^7^ cells) were lysed in RIPA buffer containing protease and phosphatase inhibitors. The lysates were incubated with anti-FOXQ1 antibody (Cell Signaling Technology) or control IgG overnight at 4 °C, followed by incubation with protein A/G agarose beads at 4 °C for 2 h. Immunoprecipitates were resolved by SDS-PAGE and silver stained with the Fast Silver Stain Kit (Beyotime). The gel pieces were destained and subjected to in-gel digestion using 10 ng/µL of trypsin (Sigma-Aldrich) at 37 °C for 20 h. The resultant peptides were analyzed by liquid chromatography-tandem mass spectrometry. Mass spectrum scans were performed using the Q Exactive Mass Spectrometer (Thermo Fisher Scientific). The raw mass spectrum data were processed using Proteome Discoverer 2.1 (Thermo Fisher Scientific) and submitted to MASCOT 2.6 (Matrix Science, London, UK) for peptide and protein identification using UniProt database (*Homo sapiens*). False discovery rate < 0.01 was used as filtering criteria for the identified proteins.

### Luciferase reporter assay

TOPFlash/FOPFlash reporter assay was used to assess β-catenin-dependent transcriptional activity [23]. In brief, TNBCs were co-transfected with TOPFlash or FOPFlash luciferase reporter constructs, together with the pRL-TK *Renilla* luciferase plasmid. Similarly, a STAT3 luciferase reporter plasmid and pRL-TK were co-transfected into TNBC cells using Lipofectamine 3000. Twenty-four hours after transfection, luciferase activities were determined using the Dual-Luciferase Reporter Assay System (Promega, Madison, WI, USA).

### Clinical samples

Tumor specimens and adjacent normal breast tissues were obtained from 105 TNBC patients who underwent surgery between January 2013 and July 2014. Clinicopathologic characteristics of the TNBC patients included are shown in Supplementary Table S2. All the patients were diagnosed with TNBC. Part of the tissue samples obtained were snap frozen to preserve RNA integrity. We excluded the patients who received neoadjuvant treatments or had a history of other malignant diseases. Written informed consent for research was obtained from each patient. This study was approved by the Ethics Committee of China Medical University.

### Statistical analysis

All data are presented as the mean ± standard deviation (SD). Statistical analyses were performed using the Student’s *t* test and one-way analysis of variance followed by Tukey’s post-hoc test. Correlation of RAPH1-i3 expression with clinicopathologic parameters was assessed using the chi-square test. Disease-free survival (DFS) was defined from the date of surgery to the date of first recurrence (local or distant) or date of the last follow-up. The survival curve was plotted by the Kaplan–Meier method and analyzed by the log-rank test. *P* < 0.05 was considered as statistically significant.

## Results

### FOXQ1 is essential for the radioresistance of TNBC cells

**Fig. 1:**
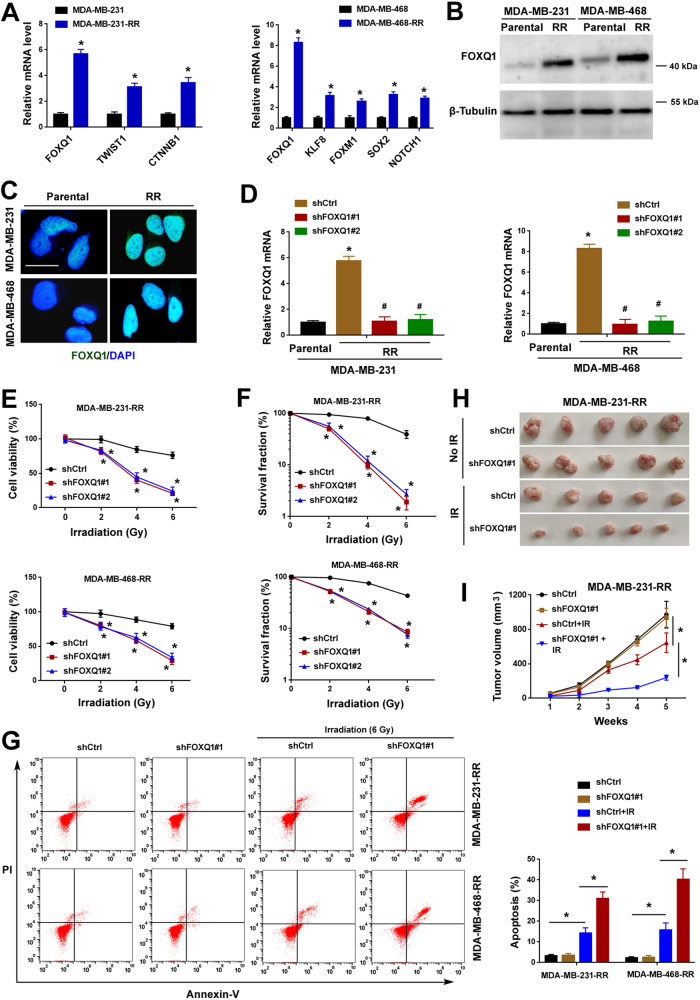
FOXQ1 is essential for the radioresistance of TNBC cells. **A** Relative mRNA expression levels of indicated genes in radioresistant and parental MDA-MB-231 and MDA-MB-468 cells. Data are presented as fold change ± SD of three independent experiments. ^*^P < 0.05 by the Student’s *t* test. **B** Western blot analysis of FOXQ1 levels in radioresistant and parental cells. **C** Immunofluorescence analysis of FOXQ1 expression (green) in radioresistant and parental cells. Nuclei were counterstained by DAPI (blue). Scale bar, 20 µm. **D** Relative mRNA expression levels of FOXQ1 in radioresistant cells transfected with indicated shRNAs. Data are presented as fold change ± SD of three independent experiments. *P < 0.05 *vs*^.^ Parental cells, ^#^*P* < 0.05 vs. the shCtrl group; one-way analysis of variance followed by Tukey’s post-hoc test. **E** Radioresistant TNBC cells transfected with indicated shRNAs were exposed to different doses of irradiation and measured for viability. *P < 0.05 vs. the shCtrl group; one-way analysis of variance followed by Tukey’s post-hoc test. **F** Clonogenic survival assays performed in radioresistant TNBC cells transfected with indicated shRNAs. *P < 0.05 vs. the shCtrl group; one-way analysis of variance followed by Tukey’s post-hoc test. **G** Transfected cells were treated with or without irradiation (IR) and subjected to apoptosis analysis by flow cytometry using annexin-v and propidium iodide (PI). Bar graphs (right panel) showing quantitative results of apoptosis assay. *P < 0.05 by one-way analysis of variance followed by Tukey’s post-hoc test. **H**, **I** MDA-MB-231-RR cells transfected with indicated shRNAs were injected into nude mice and treated with or without irradiation (IR). After 5 weeks, the tumors from each group of mice were excised and photographed (**H**). Tumor volumes were measured every week (**I**). *P < 0.05 by one-way analysis of variance followed by Tukey’s post^-^hoc test; *n* = 5 for each group.

To get insights into the relationship between EMT and TNBC radioresistance, we examined a set of 44 EMT-related transcription factors in parental and radioresistant TNBC cell lines. These transcription factors listed in Supplementary Table S3 have been proven to drive EMT in multiple cancer types [22, 23]. Compared with parental cells, MDA-MB-231-RR and MDA-MB-468-RR cells exhibited significant upregulation of 3 and 5 EMT-related genes, respectively (Fig. 1A). As FOXQ1 was consistently upregulated in both the radioresistant TNBC cell lines, we focused on this transcription factor in the following experiments. Consistently, Western blot analysis indicated a marked increase in the protein level of FOXQ1 in the radioresistant TNBC cell lines (Fig. 1B). Immunofluorescence revealed that FOXQ1 localized primarily in the nucleus of TNBC cells (Fig. 1C).

Next, we checked whether FOXQ1 upregulation is required for TNBC radioresistance. Two independent shRNAs were transduced to radioresistant TNBC cells to reduce FOXQ1 expression to the level seen in parental cells (Fig. 1D). Silencing of FOXQ1 significantly compromised cell survival in the presence of irradiation (2 Gy or a higher dose), but yielded little effect in the absence of irradiation (Fig. 1E). Clonogenic survival assays confirmed that FOXQ1 knockdown restored radiosensitivity in MDA-MB-231-RR and MDA-MB-468-RR cells (Fig. 1F). In addition, apoptosis analysis indicated that FOXQ1 depletion enhanced apoptosis in MDA-MB-231-RR and MDA-MB-468-RR cells after irradiation at 6 Gy (Fig. 1G). To validate the in vitro findings, we established xenograft tumor models by injecting MDA-MB-231-RR cells transduced with indicated shRNAs into nude mice. The tumor-bearing mice received irradiation treatment or not. Notably, knockdown of FOXQ1 significantly potentiated tumor growth control by irradiation treatment, but did not affect tumor growth without irradiation (Fig. 1H, I). Collectively, these data indicate that FOXQ1 is required for the maintenance of radioresistance in TNBC cells.

### FOXQ1 associates with RAPH1 in radioresistant TNBC cells

**Fig. 2:**
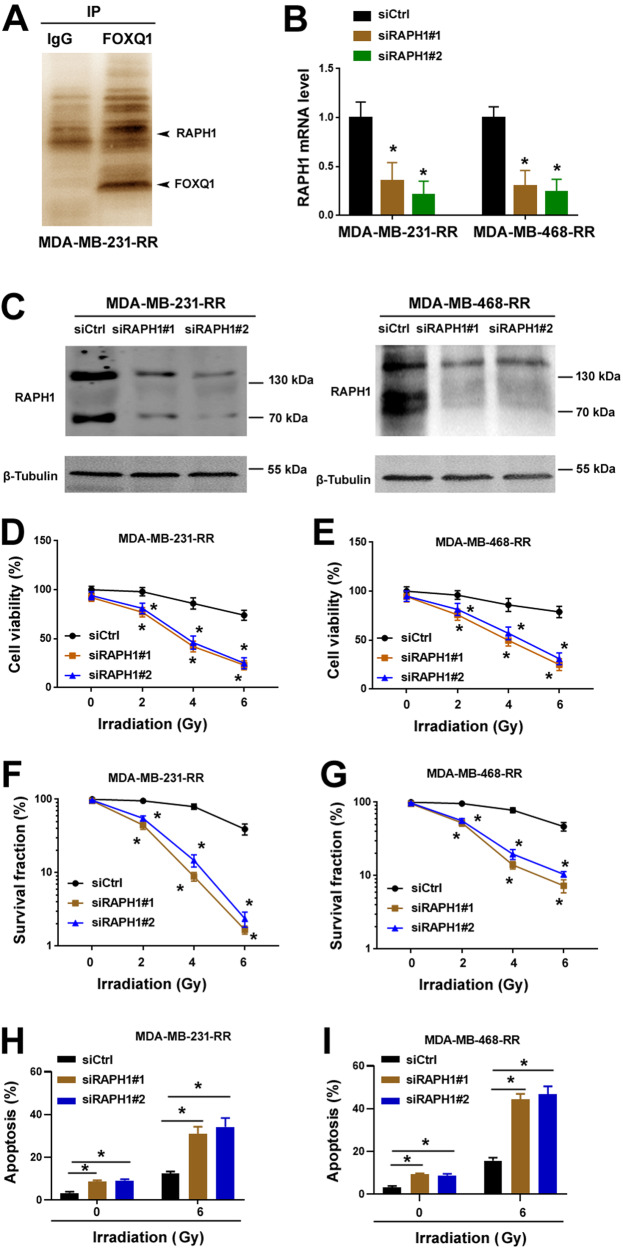
FOXQ1 associates with RAPH1 in radioresistant TNBC cells. **A** Silver staining of proteins after immunoprecipitation with control IgG or anti-FOXQ1 antibody. **B** Relative mRNA expression levels of RAPH1 in radioresistant cells transfected with indicated siRNAs. Data are presented as fold change ± SD of three independent experiments. *P < 0.05 *vs*. the siCtrl group; one-way analysis of variance followed by Tukey’s post-hoc test. **C** Western blot analysis of RAPH1 in radioresistant cells transfected with indicated siRNAs. **D**, **E** Radioresistant TNBC cells transfected with indicated siRNAs were exposed to different doses of irradiation and measured for viability. *P < 0.05 vs^.^ the siCtrl group; one-way analysis of variance followed by Tukey’s post-hoc test. **F**, **G** Clonogenic survival assays performed in radioresistant TNBC cells transfected with indicated siRNAs. *P < 0.05 vs. the siCtrl group; one-way analysis of variance followed by Tukey’s post-hoc test. **H**, **I** Apoptosis analysis by flow cytometry using annexin-v and propidium iodide. *P < 0.05 by one-way analysis of variance followed by Tukey’s post-hoc test.

Next, we investigated whether FOXQ1 upregulation is sufficient to induce the radioresistance in TNBC cells. FOXQ1 was ectopically expressed in a panel of TNBC cell lines including MDA-MB-231, MDA-MB-436, and MDA-MB-468 (Supplementary Fig. S1). Overexpression of FOXQ1 alone did not significantly increase TNBC cell survival upon irradiation treatment (Supplementary Fig. S1). We speculated that FOXQ1-mediated radioresistance might require other protein partners. To search for FOXQ1 co-factors that are involved in TNBC radioresistance, we performed immunoprecipitation experiments in MDA-MB-231-RR cells using an anti-FOXQ1 antibody. The FOXQ1 immunoprecipitate was analyzed by silver staining (Fig. 2A) and mass spectrometry. RAPH1 was one of the top candidates for interaction with FOXQ1, with peptide-spectrum matches of 85 (Supplementary Table S4). We focused on RAPH1 because of its abnormal subcellular localization. As an actin cytoskeleton modulator, RAPH1 has been known to have a cytoplasmic expression pattern [24]. Our detection of RAPH1 in FOXQ1 immunoprecipitates suggested the localization of RAPH1 in the nucleus. Most importantly, knockdown of RAPH1 increased radiosensitivity and apoptosis in MDA-MB-231-RR and MDA-MB-468-RR cells (Fig. 2B–I), which were similar to the findings seen in FOXQ1-depleted radioresistant TNBC cells (Fig. 1E–G). These data suggest that FOXQ1-mediated radioresistance may be associated with its interaction with RAPH1.

### Nuclear accumulation of the RAPH1-i3 isoform in radioresistant TNBC cells

**Fig. 3:**
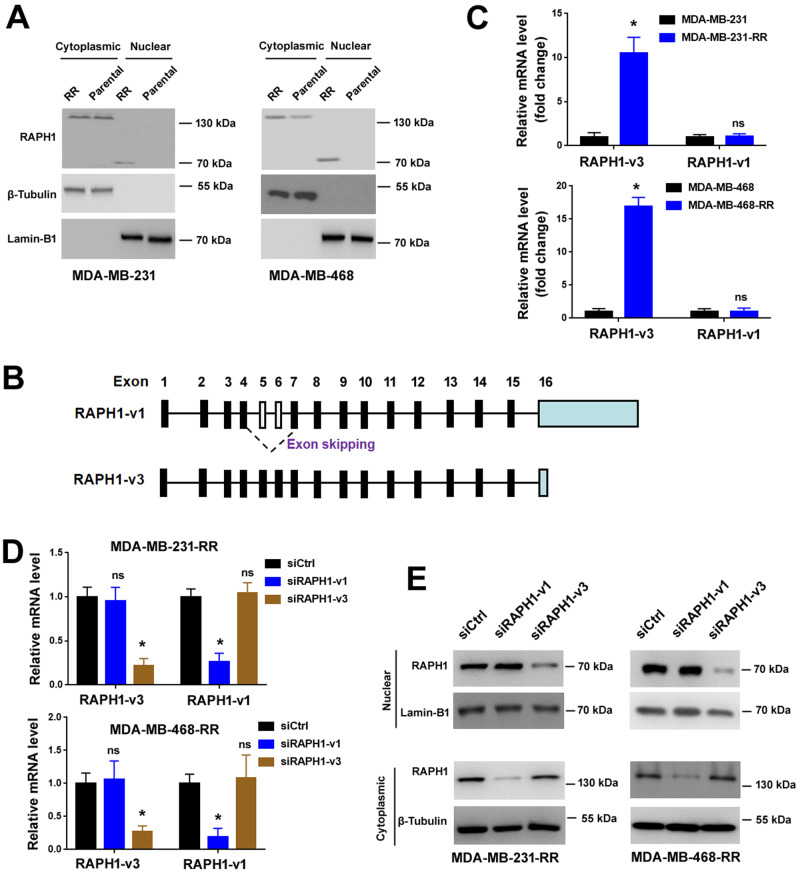
Nuclear accumulation of the RAPH1-i3 isoform in radioresistant TNBC cells. **A** Western blot analysis of RAPH1 levels in nuclear and cytoplasmic lysates from radioresistant and parental TNBC cells. **B** Schematic of *RAPH1* spliced transcripts (RAPH1-v1 and -v3). **C** Relative levels of RAPH1-v1 and -v3 transcripts in radioresistant and parental TNBC cells. Data are presented as fold change ± SD of three independent experiments. *P < 0.05 by the Student’s *t* test. ns indicates no significance. **D** Relative levels of RAPH1-v1 and -v3 transcripts in radioresistant TNBC cells transfected with indicated siRNAs. *P < 0.05 *vs*^.^ the siCtrl group; one-way analysis of variance followed by Tukey’s post-hoc test. ns indicates no significance. **E** Western blot analysis of RAPH1 levels in nuclear and cytoplasmic lysates from radioresistant TNBC cells transfected with indicated siRNAs.

To validate the nuclear accumulation of RAPH1 in radioresistant TNBC cells, we performed nuclear/cytoplasmic fractionation assays. While cytoplasmic RAPH1 was present in both radioresistant and parental TNBC cells, nuclear RAPH1 was only detected in radioresistant TNBC cells (Fig. 3A). Most interestingly, the nuclear RAPH1 isoform (~70 kDa) was shorter than the cytoplasmic RAPH1 isoform (~150 kDa). Through alternative splicing, the *RAPH1* gene produces 2 main transcript variants, i.e., RAPH1-v1 (NM_213589) and -v3 (NM_203365). RAPH1-v1 has a longer exon16 than RAPH1-v3, but lacks exon5 and exon6, which are present in RAPH1-v3 (Fig. 3B). RAPH1-v1 encodes an isoform of 1250 aa, while RAPH1-v3 yields a shorter isoform of 644 aa. The RAPH1 isoforms 1 and 3 (named RAPH1-i1 and -i3, respectively) share a common N-terminal region and a variable C-terminus. Therefore, the cytoplasmic and nuclear RAPH1 isoforms seen in radioresistant TNBC cells might be translated from RAPH1-v1 and -v3, respectively. Isoform-specific qRT-PCR analysis showed that radioresistant TNBC cells expressed greater levels of RAPH1-v3 than parental cells (Fig. 3C). In contrast, the level of RAPH1-v1 was comparable between radioresistant and parental TNBC cells. These results suggested that nuclear RAPH1 observed in radioresistant TNBC cells was encoded by RAPH1-v3. To validate this, we depleted RAPH1-v3 using specific siRNAs (Fig. 3D), which selectively target the unique sequences of RAPH1-v3. Intriguingly, knockdown of RAPH1-v3 but not RAPH1-v1 depleted nuclear RAPH1 in radioresistant TNBC cells (Fig. 3E). Taken together, radioresistant TNBC cells show abundant expression of RAPH1-v3 and nuclear accumulation of the RAPH1-i3 isoform.

### RAPH1-i3 cooperates with FOXQ1 to promote growth, migration, and radioresistance in breast cancer cells

**Fig. 4:**
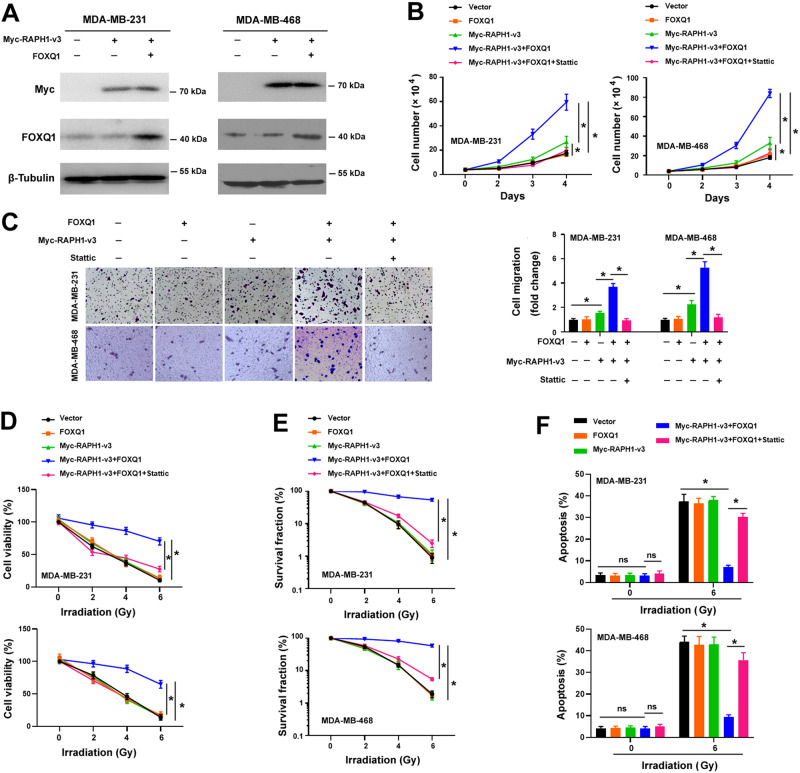
RAPH1-i3 cooperates with FOXQ1 to promote growth, migration, and radioresistance in parental TNBC cells. **A** Western blot analysis of Myc-tagged RAPH1-v3 and FOXQ1 in TNBC cells transfected with indicated constructs. **B** Cell proliferation assay in MDA-MB-231 and MDA-MB-468 cells transfected with indicated constructs and treated with Stattic (2 μM). *P < 0.05 by one-way analysis of variance followed by Tukey’s post-hoc test. **C** Transwell migration assay in MDA-MB-231 and MDA-MB-468 cells transfected with indicated constructs and treated with Stattic (2 μM). *Left*, representative images of migrated cells. *P < 0.05 by one-way analysis of variance followed by Tukey’s post-hoc test. **D** TNBC cells with indicated treatments were exposed to different doses of irradiation and measured for viability. *P < 0.05 by one-way analysis of variance followed by Tukey’s post-hoc test. **E** Clonogenic survival assays performed in TNBC cells with indicated treatments. *P < 0.05 by one-way analysis of variance followed by Tukey’s post-hoc test. **F** Apoptosis analysis by flow cytometry using annexin-v and propidium iodide. *P < 0.05 by one-way analysis of variance followed by Tukey’s post-hoc test. ns indicates no significance.

Next, we investigated the biological role of RAPH1-i3 in TNBC. We constructed the Myc-tagged RAPH1-v3-expressing plasmid and transfected it to parental MDA-MB-231 and MDA-MB-468 cells. Overexpression of RAPH1-i3 was confirmed by Western blot analysis (Fig. 4A). Interestingly, enforced expression of RAPH1-i3 promoted TNBC cell proliferation and migration (Fig. 4B, C). When FOXQ1 was coexpressed with RAPH1-i3, the promotion of cell proliferation and migration was further enhanced (Fig. 4A–C). Although overexpression of RAPH1-i3 alone did not significantly induce radioresistance in parental TNBC cells, co-expression of RAPH1-i3 and FOXQ1 rendered parental TNBC cells resistant to irradiation treatment (Fig. 4D–F). We also evaluated the cooperation between FOXQ1 and RAPH1-i3 in non-TNBC cells. Of note, co-expression of RAPH1-i3 and FOXQ1 promoted cell proliferation and increased radioresistance in 2 estrogen receptor-positive breast cancer cell lines, MCF7 and T47D (Supplementary Fig. S2). These results suggest that the cooperation between RAPH1-i3 and FOXQ1 is important for breast cancer progression.

### RAPH1-i3 interacts with FOXQ1 to boost STAT3-dependent transcriptional activity

**Fig. 5:**
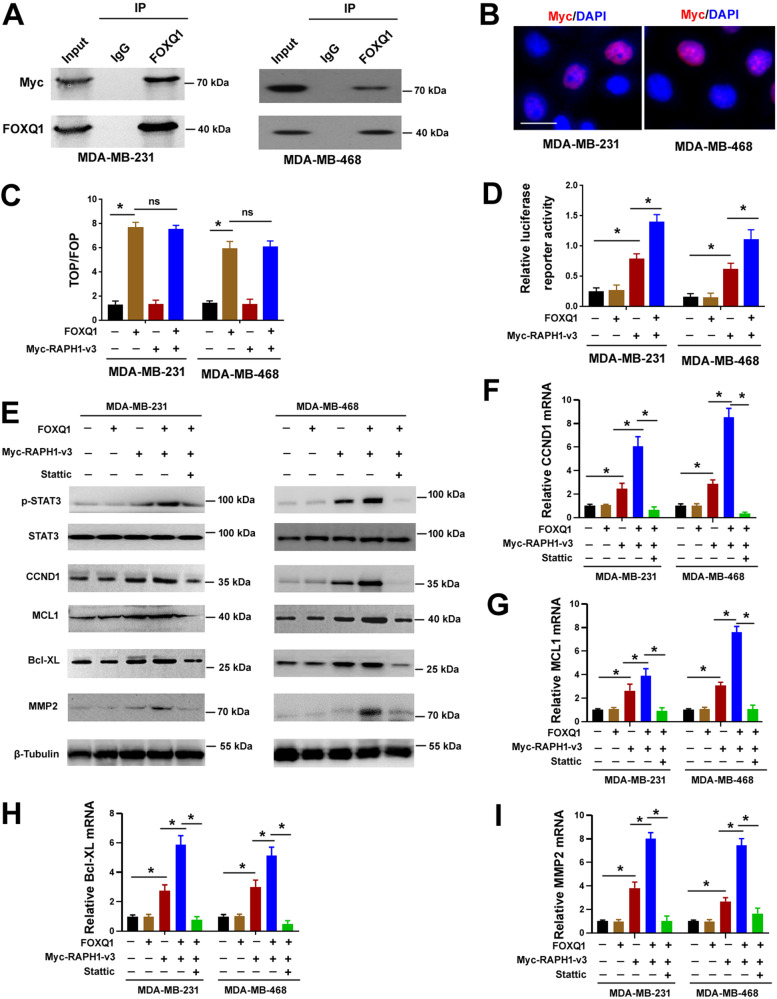
RAPH1-i3 interacts with FOXQ1 to boost STAT3-dependent transcriptional activity. **A** TNBC cells were transfected with Myc-tagged RAPH1-i3 and subjected to co-immunoprecipitation assays using an anti-FOXQ1 antibody. **B** Immunofluorescent staining of exogenously expressed Myc-tagged RAPH1-i3 (red). Nuclei were counterstained by DAPI (blue). Scale bar, 20 µm. **C** TOPFlash/FOPFlash reporter assays were performed to evaluate β-catenin-dependent transcriptional activity. *P < 0.05 by one-way analysis of variance followed by Tukey’s post-hoc test. ns indicates no significance. **D** STAT3 specific luciferase reporter plasmid was co-transfected with FOXQ1 and RAPH1-i3. Relative STAT3 luciferase activity was measured 24 h after transfection. *P < 0.05 by one-way analysis of variance followed by Tukey’s post-hoc test. **E** Western blot analysis of phosphorylated STAT3 (p-STAT3), STAT3, CCND1, MCL1, Bcl-XL, and MMP2 in TNBC cells transfected with indicated constructs and treated with Stattic (2 μM). **F**–**I** Relative mRNA expression levels of indicated genes in TNBC cells transfected with indicated constructs and treated with Stattic (2 μM). Data are presented as fold change ± SD of three independent experiments. *P < 0.05 by one-way analysis of variance followed by Tukey’s post-hoc test.

Next, we sought to determine the mechanism by which RAPH1-i3 and FOXQ1 regulate the aggressiveness of breast cancer cells. Co-immunoprecipitation assays using an anti-FOXQ1 antibody demonstrated that exogenously expressed RAPH1-i3 was associated with FOXQ1 in TNBC cells (Fig. 5A). Immunofluorescent staining indicated that RAPH1-i3 was localized in the nucleus (Fig. 5B), suggesting its potential role in the regulation of gene expression. FOXQ1 overexpression or knockdown did not affect the RAPH1-i3 protein expression in TNBC cells (Supplementary Fig. S3). FOXQ1 has been reported to regulate Wnt/β-catenin signaling, a key pathway involved in cancer progression [25]. Using the TOPflash β-catenin/TCF reporter system, we found that FOXQ1 overexpression increased β-catenin-dependent transcriptional activity (Fig. 5C). However, co-expression of RAPH1-i3 did not boost FOXQ1-induced β-catenin transcriptional activity. Thus, other signaling pathways may mediate the effect of RAPH1-i3 and FOXQ1 cooperation on TNBC cells. Since activation of STAT3 signaling promotes TNBC radioresistance and metastasis [5, 21], we checked whether RAPH1-i3 and FOXQ1 could regulate STAT3 signaling activation. STAT3 luciferase reporter assays demonstrated that co-expression of RAPH1-i3 and FOXQ1 significantly promoted STAT3 transcriptional activity compared with overexpression of RAPH1-i3 alone (Fig. 5D). The amount of phosphorylated STAT3 (Tyr705) increased in TNBC cells upon overexpression of RAPH1-i3 or co-expression of RAPH1-i3 and FOXQ1 (Fig. 5E). However, overexpression of FOXQ1 alone had no impact on STAT3 activation status. Analysis of STAT3 target genes showed that co-expression of RAPH1-i3 and FOXQ1 significantly increased the expression of CCND1, MCL1, Bcl-XL, and MMP2 (Fig. 5E–I). We next investigated the effect of RAPH1-i3 and FOXQ1 co-expression on EMT markers. As shown in Supplementary Fig. S4, FOXQ1 overexpression induced the expression of vimentin and Twist1 without affecting E-cadherin and ZEB1. FOXQ1-dependent induction of vimentin and Twist1 was not augmented when RAPH1-i3 was co-expressed.

To validate whether the aggressive phenotype induced by the RAPH1-i3 and FOXQ1 co-expression is dependent on STAT3 activation, we inhibited STAT3 activity in TNBC cells transfected with RAPH1-i3 and FOXQ1 using the STAT3 phosphorylation inhibitor Stattic. Notably, the Stattic treatment effectively blocked the proliferation, migration, and radioresistance induced by RAPH1-i3 and FOXQ1 co-expression (Fig. 4B–F), which was accompanied by inhibition of STAT3 target gene expression (Fig. 5E–I). Taken together, RAPH1-i3 and FOXQ1 co-expression promotes TNBC progression and radioresistance by inducing STAT3 signaling activation.

### Depletion of RAPH1-v3 and FOXQ1 reverses TNBC radioresistance by suppressing STAT3 signaling

**Fig. 6:**
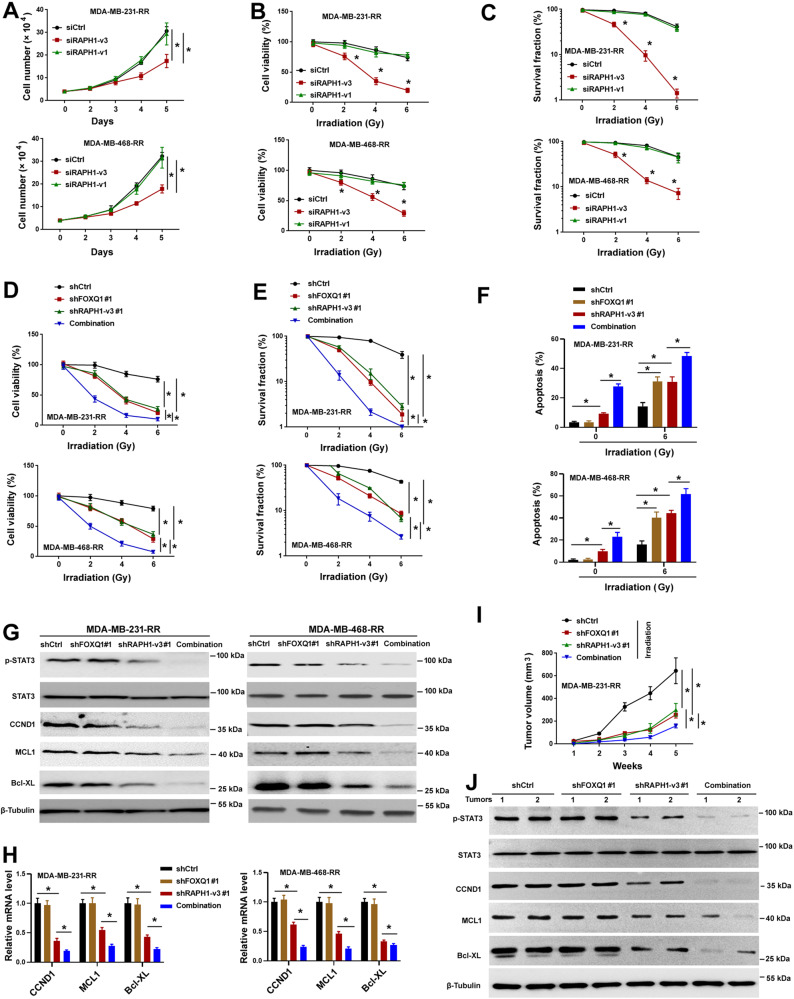
Depletion of RAPH1-i3 enhances the radiosensitivity of radioresistant TNBC cells by suppressing STAT3 signaling. **A** Cell proliferation assays performed in radioresistant TNBC cells transfected with indicated siRNAs. *P < 0.05 by one-way analysis of variance followed by Tukey’s post-hoc test. **B** Radioresistant TNBC cells transfected with indicated siRNAs were exposed to different doses of irradiation and measured for viability. *P < 0.05 by one-way analysis of variance followed by Tukey’s post-hoc test. **C** Clonogenic survival assays performed in radioresistant TNBC cells transfected with indicated siRNAs. *P < 0.05 by one-way analysis of variance followed by Tukey’s post-hoc test. **D** Measurement of the viability of radioresistant TNBC cells transfected with control shRNA (shCtrl), shFOXQ1#1, shRAPH1-v3#1, or shFOXQ1#1 plus shRAPH1-v3#1 (combination). *P < 0.05 by one-way analysis of variance followed by Tukey’s post-hoc test. **E** Clonogenic survival assays performed in radioresistant TNBC cells transfected with indicated shRNAs. *P < 0.05 by one-way analysis of variance followed by Tukey’s post-hoc test. **F** Analysis of apoptosis in radioresistant TNBC cells transfected with indicated shRNAs. *P < 0.05 by one-way analysis of variance followed by Tukey’s post-hoc test. **G** Western blot analysis of phosphorylated STAT3 (p-STAT3), STAT3, CCND1, MCL1, and Bcl-XL in radioresistant TNBC cells transfected with indicated shRNAs. **H** Relative mRNA expression levels of indicated genes in radioresistant TNBC cells transfected with indicated shRNAs. Data are presented as fold change ± SD of three independent experiments. *P < 0.05 by one-way analysis of variance followed by Tukey’s post^-^hoc test. **I**, **J** MDA-MB-231-RR cells transfected with indicated shRNAs were injected into nude mice and then treated with irradiation 1 week after cell injection. **I** Tumor volumes were measured every week (*n* = 5 for each group). *P < 0.05 by one-way analysis of variance followed by Tukey’s post-hoc test. **J** Western blot analysis of p-STAT3, STAT3, CCND1, MCL1, and Bcl-XL protein levels in 2 representative xenograft tumors from each group.

Given the abundant expression of RAPH1-i3 in radioresistant TNBC cells, we investigated the effect of depletion of RAPH1-i3 on TNBC cell radiosensitivity. Targeted reduction of RAPH1-v3 abrogated the growth of MDA-MB-231-RR and MDA-MB-468-RR cells (Fig. 6A). Moreover, the radiosensitivity of radioresistant TNBC cells was increased when RAPH1-v3 was depleted (Fig. 6B, C). However, depletion of RAPH1-v1 did not phenocopy the effects of RAPH1-v3 silencing in the radioresistant TNBC cells. These results indicate that RAPH1-i3 is required for TNBC growth and radioresistance.

Since FOXQ1 and RAPH1-i3 play an important role in radioresistant TNBC cells, we assessed if co-knockdown of FOXQ1 and RAPH1-i3 could reverse TNBC radioresistance both in vitro and in vivo. Two independent shRNAs were designed to target RAPH1-v3, yielding effective knockdown of RAPH1-v3 but not FOXQ1 in MDA-MB-231-RR and MDA-MB-468-RR cells (Supplementary Fig. S5). Co-delivery of shRAPH1-v3 and shFOXQ1 led to simultaneous depletion of RAPH1-v3 and FOXQ1 and remarkably restored radiosensitivity in radioresistant TNBC cells (Fig. 6D–F). At the molecular level, co-knockdown of RAPH1-v3 and FOXQ1 inhibited STAT3 phosphorylation and the expression of STAT3 target genes (Fig. 6G, H). In vivo studies further demonstrated that the xenograft tumors with depletion of both RAPH1-v3 and FOXQ1 showed more sensitive to irradiation compared to RAPH1-v3- or FOXQ1-depleted xenograft tumors (Fig. 6I). Western blot analysis of the xenograft tumors confirmed the decrease in STAT3 phosphorylation and CCND1, MCL1, Bcl-XL expression in the RAPH1-v3 and FOXQ1 co-depletion group (Fig. 6J). These results suggest that targeting RAPH1-v3 and FOXQ1 impairs TNBC growth and radioresistance through the STAT3 pathway.

### Upregulation of RAPH1-i3 predicts poor prognosis in TNBC

**Fig. 7:**
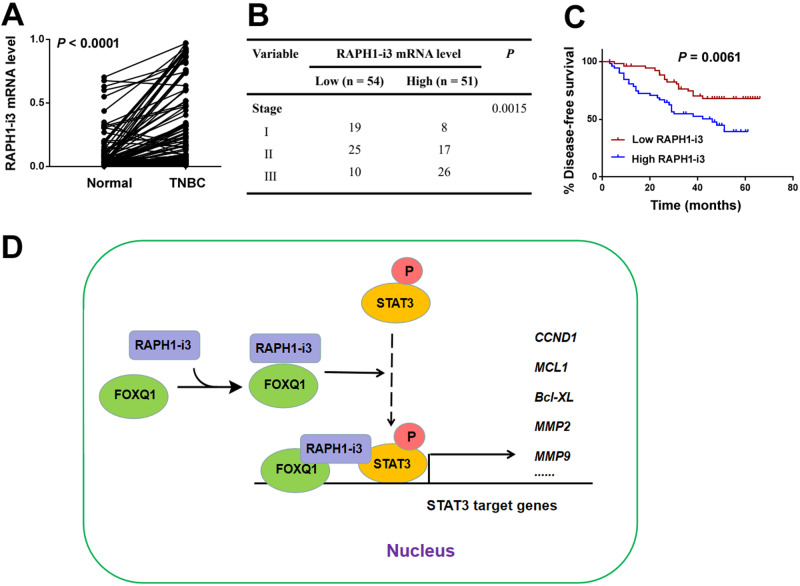
Upregulation of RAPH1-i3 predicts poor prognosis in TNBC. **A** Relative levels of RAPH1-i3 transcripts in 105 paired TNBC and adjacent normal breast tissues. **B** Analysis of the relationship between RAPH1-i3 levels and tumor stage in TNBC patients. **C** Kaplan–Meier survival curves were plotted to analyze the relationship between RAPH1-i3 levels and disease-free survival of TNBC patients. **D** Schematic diagram of the molecular mechanism by which RAPH1-i3 interacts with FOXQ1 to promote tumor progression and radioresistance of TNBC through activation of STAT3 signaling.

We also investigated the expression and clinical value of RAPH1-i3 in TNBC. Compared with adjacent normal breast tissues, TNBC samples showed significantly increased levels of RAPH1-i3 transcripts (*P* < 0.0001; Fig. 7A). Based on the median level of RAPH1-i3, TNBC patients were divided into high and low RAPH1-i3 groups. Higher RAPH1-i3 expression was significantly correlated with advanced tumor stage of TNBC patients (*P* = 0.0015; Fig. 7B). In addition, the high RAPH1-i3 group had significantly reduced DFS compared with the low RAPH1-i3 group (*P* = 0.0061; Fig. 7C). Collectively, RAPH1-i3 upregulation is associated with poor prognosis in TNBC patients.

## Discussion

Compelling evidence establishes a link between EMT and chemoradioresistance [26–29]. The EMT transcription factor ZEB1 interacts with USP7 to stabilize CHK1 and contributes to radioresistance [15]. Another EMT transcription factor FOXM1 induces docetaxel resistance in castration-resistant prostate cancer cells [30]. In this study, we profiled the expression of the 44 known EMT transcription factors in radioresistant and parental TNBC cells. Our data show that FOXQ1 is significantly upregulated in radioresistant TNBC cells, suggesting its involvement in TNBC radioresistance. Previous studies have reported that FOXQ1 can activate EMT and promote metastasis and chemoresistance in breast cancer [18, 31]. Consistently, our results indicate that FOXQ1 plays a pivotal role in the maintenance of radioresistance in TNBC cells. We find that depletion of FOXQ1 increases the radiosensitivity of TNBC cells. However, overexpression of FOXQ1 alone is not sufficient to induce the radioresistance in TNBC cells. Mitchell et al. [31] demonstrated that FOXQ1 recruits the MLL/KMT2 histone methyltransferase complex to regulate gene expression. Meng et al. [18] reported that FOXQ1 can interact with Twist1 to modulate downstream target genes. We reasoned that FOXQ1 might need co-factors to drive the radioresistance in TNBC.

Notably, we find that FOXQ1 associates with RAPH1 in the nucleus of radioresistant TNBC cells. RAPH1 is known to have a cytoplasmic localization [24]. The nuclear RAPH1 isoform is shorter than the cytoplasmic isoform and may be encoded by the alternatively spliced transcript, RAPH1-v3. When RAPH1-v3 was depleted, the nuclear RAPH1 isoform disappeared. Moreover, enforced expression of RAPH1-v3 led to nuclear accumulation of RAPH1. Compared with parental TNBC cells, radioresistant TNBC cells expressed higher levels of RAPH1-v3. These results suggest that the nuclear isoform of RAPH1 might contribute to TNBC radioresistance. RAPH1 has been reported to mediate stiffness-induced cell cyclin expression and cell proliferation [32]. RAPH1 can form a complex with integrins to guide cell migration [33]. RAPH1 can also cooperate with oncogenic Ras to drive the development of glial tumors [34]. In breast cancer cells, RAPH1 has the capacity to promote three-dimensional (3D) cancer cell invasion through interaction with actin-elongating Ena/VASP proteins and the Scar/WAVE complex [35]. Consistently, our data indicate that the nuclear isoform of RAPH1 stimulates TNBC cell proliferation and migration, which can be enhanced by co-expression of FOXQ1. Moreover, FOXQ1 and RAPH1-i3 cooperation yields similar oncogenic outcomes in non-TNBC cells such as MCF7 and T47D cells. Taken together, these findings support the pivotal role for the nuclear isoform of RAPH1 in mediating breast cancer aggressive phenotype.

Similar to FOXQ1 knockdown, depletion of the nuclear isoform of RAPH1 restores the radiosensitivity in radioresistant TNBC cells. However, neither of them alone fails to establish radioresistance in parental TNBC cells. Most importantly, coexpression of FOXQ1 and the nuclear RAPH1 isoform renders parental TNBC cells resistant to irradiation. These results indicate the importance of FOXQ1 and nuclear RAPH1 cooperation in inducing TNBC radioresistance. Mechanistically, overexpression of nuclear RAPH1 promotes STAT3 phosphorylation and activation, which is further reinforced by coexpression of FOXQ1. Activation of STAT3 signaling has been reported to induce radioresistance in TNBC [5, 22]. Multiple STAT3 target genes including CCND1, MCL1, Bcl-XL, and MMP2 can be significantly induced when nuclear RAPH1 and FOXQ1 are overexpressed together. Both MCL1 and Bcl-XL are antiapoptotic genes and contribute to the radioresistance in cancer cells [22, 36]. Collectively, coexpression of nuclear RAPH1 and FOXQ1 drives TNBC radioresistance through activation of STAT3 signaling (Fig. 7D). The robustness of STAT3 activation due to overexpression of either nuclear RAPH1 or FOXQ1 alone might be not enough to initiate TNBC radioresistance. In addition, our data show that FOXQ1 overexpression enhances β-catenin-dependent transcriptional activity. Activation of the Wnt/β-catenin signaling pathway has been reported to contribute to cancer radioresistance [37]. Hence, the radioresistance induced by coexpression of nuclear RAPH1 and FOXQ1 might also involve activation of β-catenin signaling.

A previous study has reported that RAPH1 mRNA upregulation is correlated with poor prognosis in breast cancer [38]. Such overexpression is associated with high proliferation index and triple negative phenotype. In this study, we examined the clinical significance of the nuclear isoform of RAPH1. Our data show that the nuclear isoform of RAPH1 is upregulated in TNBC tissues relative to adjacent normal breast tissues. Moreover, high expression of nuclear RAPH1 is significantly associated with advanced tumor stage and reduced DFS in TNBC patients. These clinical data suggest the accumulation of the nuclear isoform of RAPH1 during TNBC progression.

However, the mechanism for the synthesis of the nuclear RAPH1 isoform remains to be clarified. Future work is needed to identify the key splicing factors responsible for the generation of the RAPH1-v3 transcript. Additionally, it deserves further investigation how the RAPH1-i3 isoform is transported to the nucleus.

In summary, we demonstrate the upregulation of the nuclear isoform of RAPH1, RAPH1-i3 in radioresistant TNBC cells. The interaction between RAPH1-i3 and FOXQ1 induces aggressiveness and radioresistance in TNBC and estrogen receptor-positive breast cancer. Overexpression of RAPH1-i3 in tumor tissues is associated with poor prognosis in TNBC patients. Targeting RAPH1-i3 and FOXQ1 may provide a therapeutic strategy for improving radioresistance in breast cancer.

### Supplementary information

Supplementary data

Original Data File

aj-checklist

## Data Availability

The data are available from the corresponding authors upon reasonable request.
