# Supplementary material for: Nuclear isoform of RAPH1 interacts with FOXQ1 to promote aggressiveness and radioresistance in breast cancer
Source: Cell Death Dis. 2023 Dec 7;14(12):803. doi: 10.1038/s41419-023-06331-9 (PMC10703867; doi:10.1038/s41419-023-06331-9)
Supplement: Supplementary file 1 — Supplementary data [file 41419_2023_6331_MOESM1_ESM.doc]

**Supplementary data**


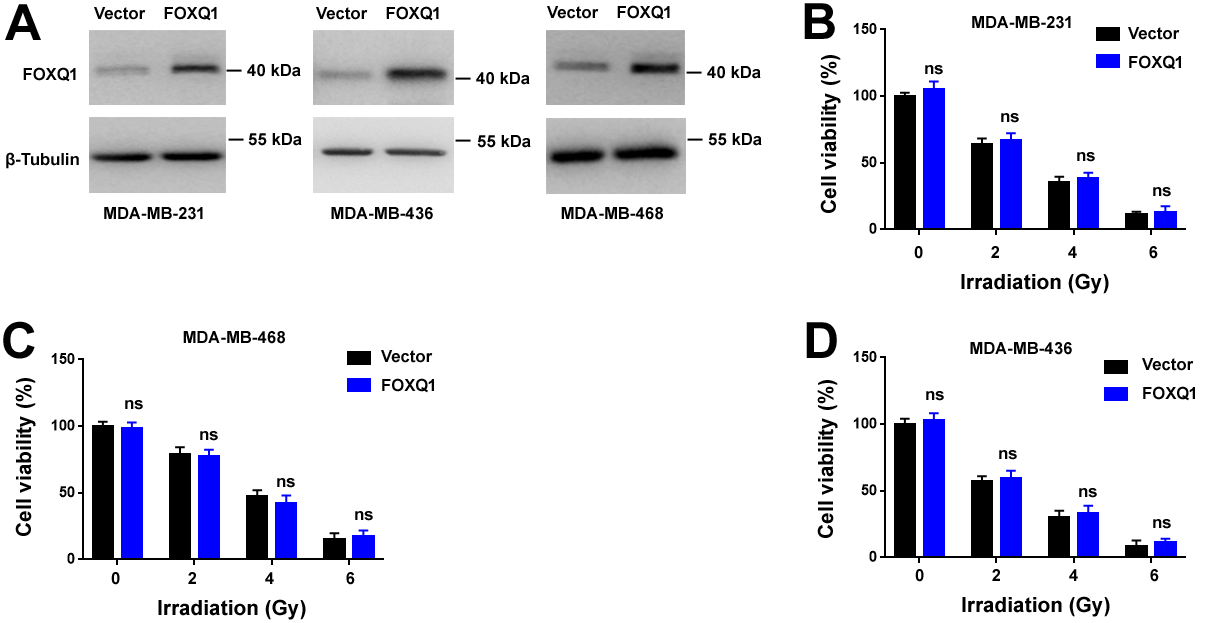


Supplementary Figure S1. Overexpression of FOXQ1 has no effect on the radiosensitivity of TNBC cells. (A) Western blot analysis of FOXQ1 in TNBC cells transfected with indicated constructs. (B-D) TNBC cells were transfected with indicated constructs and treated with different doses of irradiation. Cell viability was measured. ns indicates no significance relative to the vector group.


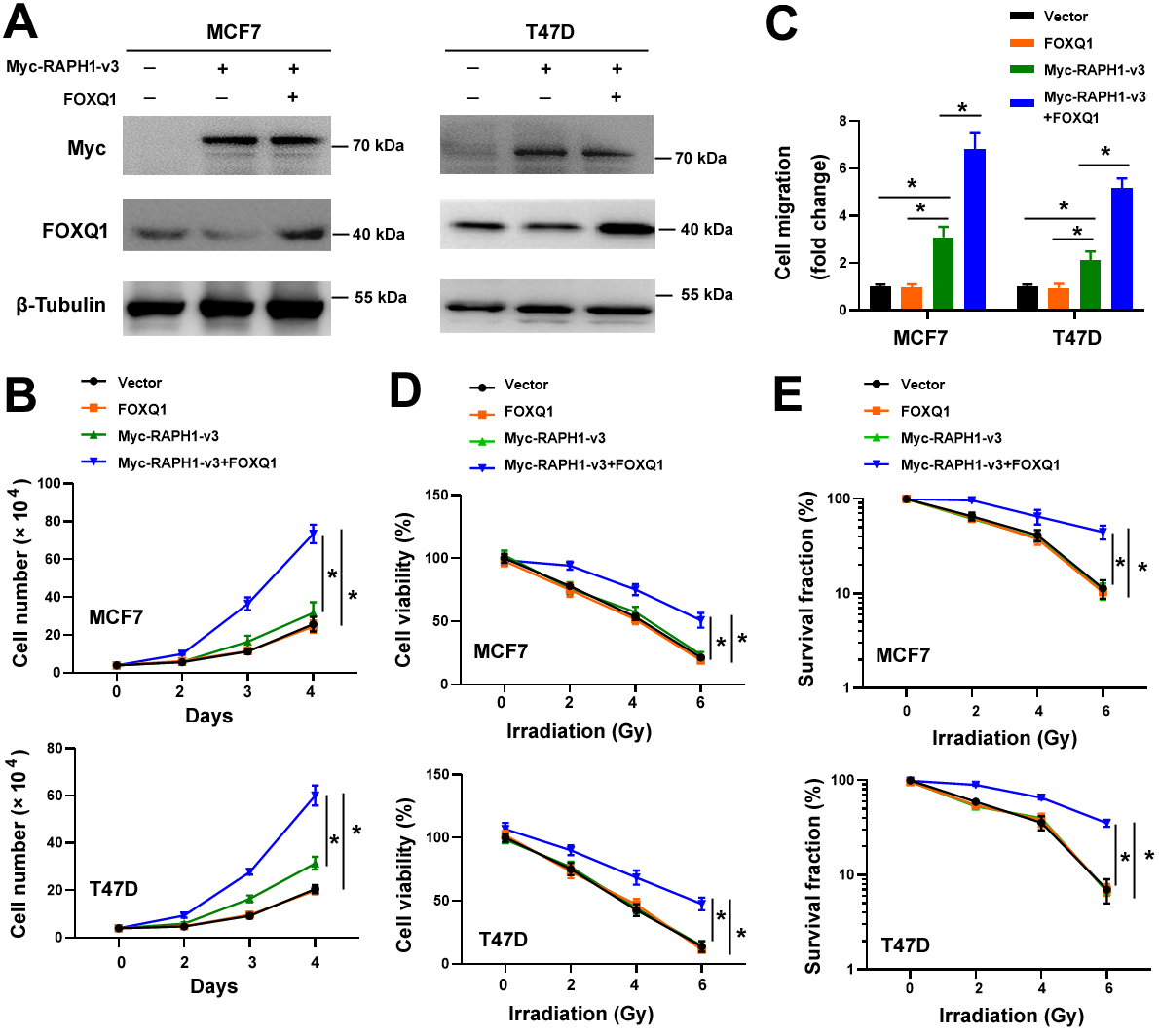


Supplementary Figure S2. Effect of FOXQ1 and RAPH1-i3 cooperation on estrogen receptor-positive breast cancer cell lines. (A) Western blot analysis of Myc-tagged RAPH1-v3 and FOXQ1 in MCF7 and T47D cells transfected with indicated constructs. (B) Cell proliferation assay in MCF7 and T47D cells transfected with indicated constructs. ^*^*P* < 0.05 by one-way analysis of variance followed by Tukey’s post-hoc test. (C) Transwell migration assay in MCF7 and T47D cells transfected with indicated constructs. ^*^*P* < 0.05 by one-way analysis of variance followed by Tukey’s post-hoc test. (D) MCF7 and T47D cells transfected with indicated constructs were exposed to different doses of irradiation and measured for viability. ^*^*P* < 0.05 by one-way analysis of variance followed by Tukey’s post-hoc test. (E) Clonogenic survival assays in MCF7 and T47D cells transfected with indicated constructs. ^*^*P* < 0.05 by one-way analysis of variance followed by Tukey’s post-hoc test.


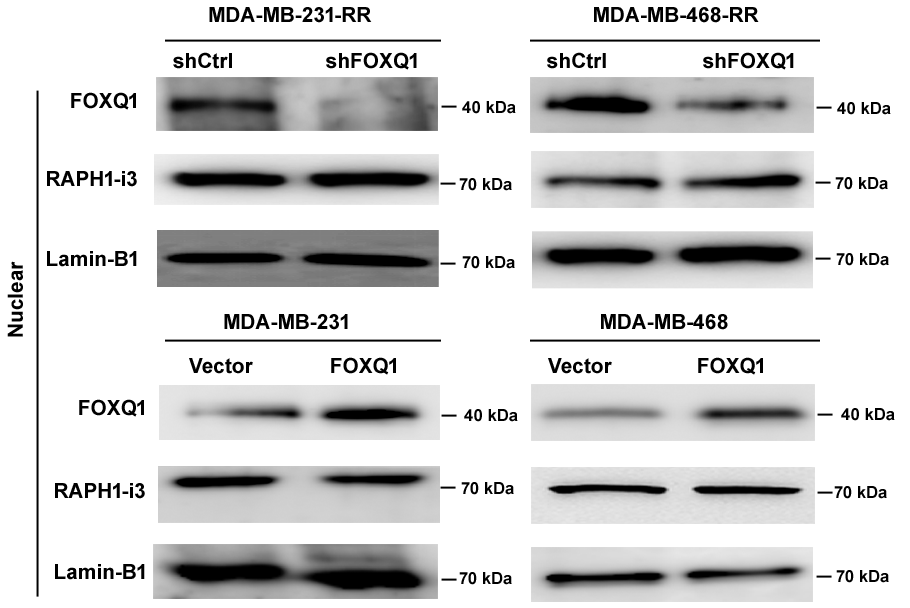


Supplementary Figure S3. Effect of FOXQ1 overexpression or knockdown on the expression of RAPH1-i3 in the nucleus.


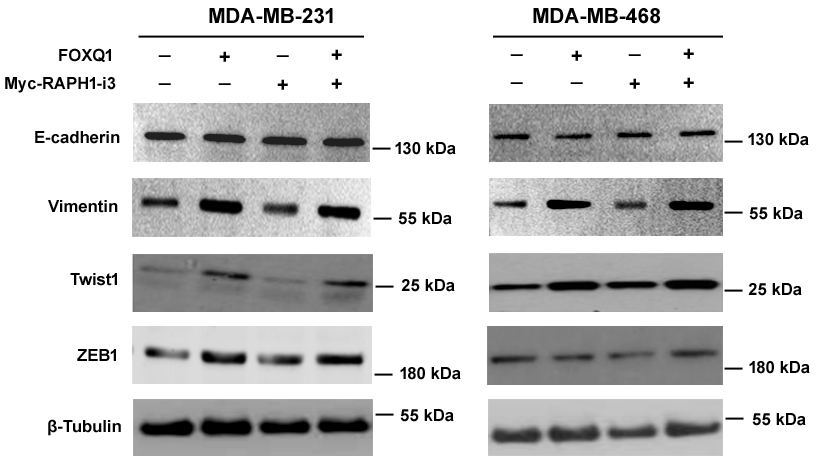


Supplementary Figure S4. Western blot analysis of EMT markers in MDA-MB-231 and MDA-MB-468 cells transfected with indicated constructs.


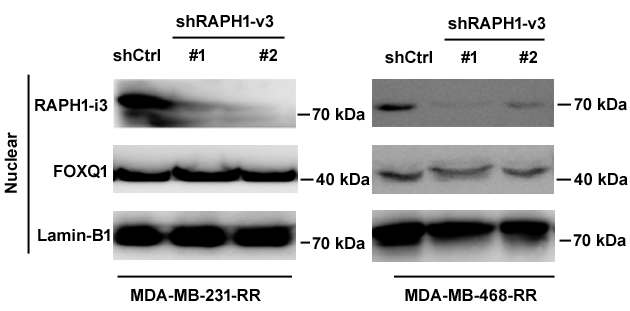


Supplementary Figure S5. Measurement of RAPH1-i3 and FOXQ1 in the nucleus after transfection with indicated shRNAs.

**Supplementary Table S1. Primers for qRT-PCR analysis**

| **Gene** | **Sequence (5′ – 3′)** |
| --- | --- |
| *TWIST1* | Forward: GCAGGACGTGTCCAGCTC |
|  | Reverse: CTGGCTCTTCCTCGCTGTT |
| *KLF8* | Forward: TCTGCAGGGACTACAGCAAG |
|  | Reverse: TCACATTGGTGAATCCGTCT |
| *FOXM1* | Forward: TGCAGCTAGGGATGTGAATCTTC |
|  | Reverse: GGAGCCCAGTCCATCAGAACT |
| *FOXQ1* | Forward: CGCGGACTTTGCACTTTGAA |
|  | Reverse: CCTGGACTAAGTTTAAATACT |
| *SOX2* | Forward: GAGCTTTGCAGGAAGTTTGC |
|  | Reverse: GCAAGAAGCCTCTCCTTGAA |
| *NOTCH1* | Forward: CGGGTCCACCAGTTTGAATG |
|  | Reverse: GTTGTATTGGTTCGGCACCAT |
| *CTNNB1* | Forward: GTGGATACCTCCCAAGTC |
|  | Reverse: GTGTAGATGGGATCTGCATG |
| *RAPH1* | Forward: GGAGCAGCTATCAGATGAAGAA |
|  | Reverse: CAAGTTGTATATGGAGAAGCGG |
| *RAPH1-v1* | Forward: GCATTGTGAGCTCCGTATTCTC |
|  | Reverse: GTGCTGTACTTGACGAACATTG |
| *RAPH1-v3* | Forward: GCATTGTGAGCTCCGTATTCTC |
|  | Reverse: CGTGTTGGTGTGAGACAATTGC |
| *CCND1* | Forward: AGGCGGAGGAGAACAAACAGA |
|  | Reverse: GGAGGGCGGATTGGAAATGAA |
| *MCL1* | Forward: GGACATCAAAAACGAAGACG |
|  | Reverse: GCAGCTTTCTTGGTTTATGG |
| *Bcl-XL* | Forward: GGAGAACGGCGGCTGGGATA |
|  | Reverse: GGCCACAGTCATGCCCGTCA |
| *MMP2* | Forward: ATTGTATTTGATGGCATCGCTC |
|  | Reverse: ATTCATTCCCTGCAAAGAACAC |
| *GAPDH* | Forward: TCTCCTCTGACTTCAACAGC |
|  | Reverse: CTGTTGCTGTAGCCAAATTCG |

**Supplementary Table S2. Clinicopathologic characteristics of the TNBC patients included in this study**

| **Characteristics** | **n = 105** |
| --- | --- |
| Age, years |  |
| Median | 51 |
| Range | 35-78 |
| Menopausal status |  |
| Yes | 65 |
| No | 40 |
| Stage |  |
| I | 27 |
| II | 42 |
| III | 36 |
| Histological grade |  |
| I-II | 35 |
| III | 70 |

**Supplementary Table S3. Expression of EMT-related genes in parental and radioresistant TNBC cells based on quantitative PCR array**

| **Gene** | **MDA-MB-231**  **(radioresistant/parental)** | **MDA-MB-468**  **(radioresistant/parental)** |
| --- | --- | --- |
| SNAIL | 1.36 | 1.02 |
| SLUG | 0.91 | 0.98 |
| ZEB1 | 1.59 | 1.08 |
| ZEB2 | 1.33 | 0.87 |
| TWIST1 | 3.12 | 1.19 |
| KLF4 | 1.20 | 1.36 |
| KLF8 | 1.14 | 3.17 |
| KLF10 | 0.89 | 0.94 |
| FOXC1 | 0.86 | 1.38 |
| FOXC2 | 1.27 | 1.19 |
| FOXK1 | 1.04 | 1.00 |
| FOXG1 | 0.98 | 1.35 |
| FOXF2 | 1.51 | 1.00 |
| FOXM1 | 1.18 | 2.58 |
| FOXQ1 | 5.69 | 8.32 |
| FOXN2 | 1.14 | 1.05 |
| FOXO3a | 1.37 | 1.10 |
| SOX4 | 0.92 | 0.99 |
| SOX9 | 0.97 | 1.08 |
| SOX11 | 1.32 | 1.21 |
| SOX2 | 1.00 | 3.25 |
| RUNX1 | 1.28 | 1.44 |
| RUNX2 | 1.03 | 1.18 |
| GATA4 | 1.42 | 1.05 |
| GATA6 | 1.64 | 1.17 |
| WT1 | 0.96 | 0.99 |
| TFAP2A | 0.93 | 1.14 |
| ZBTB38 | 1.30 | 1.37 |
| ALX1 | 0.96 | 0.98 |
| PRRX1 | 0.95 | 0.99 |
| NOTCH1 | 0.92 | 2.89 |
| E47 | 0.98 | 0.99 |
| BACH1 | 1.38 | 1.05 |
| STAT3 | 1.42 | 1.11 |
| CTNNB1 | 3.44 | 1.03 |
| TCF4 | 1.46 | 1.23 |
| SMAD2 | 0.98 | 0.94 |
| MITF | 0.97 | 1.42 |
| ESR1 | 1.19 | 1.68 |
| TCF7L1 | 0.84 | 0.97 |
| LBX1 | 0.96 | 1.44 |
| SALL4 | 1.25 | 1.00 |
| HOXA9 | 1.09 | 1.03 |
| HOXC8 | 1.18 | 0.92 |

**Supplementary Table S4. List of FOXQ1-interacting proteins identified by co-immunoprecipitation and mass spectrometry**

| **Accession** | **Gene Symbol** | **# Peptides** | **# PSMs** |
| --- | --- | --- | --- |
| Q9C009 | FOXQ1 | 8 | 118 |
| Q6P2Q9 | PRPF8 | 12 | 89 |
| Q70E73 | RAPH1 | 30 | 85 |
| Q9NX58 | LYAR | 9 | 76 |
| Q7Z406 | MYH14 | 18 | 75 |
| Q9NR30 | DDX21 | 20 | 50 |
| Q8IXJ6 | SIRT2 | 13 | 47 |
| Q7L2E3 | DHX30 | 10 | 42 |
| Q9UHB6 | LIMA1 | 19 | 27 |
| P23246 | SFPQ | 9 | 24 |
| Q9HCS7 | XAB2 | 16 | 23 |
| P49848 | TAF6 | 7 | 22 |
| P09874 | PARP1 | 10 | 22 |
| J3KTA4 | DDX5 | 13 | 21 |
| Q14974 | KPNB1 | 10 | 12 |
| Q9NU22 | MDN1 | 4 | 10 |
| Q13573 | SNW1 | 6 | 6 |
| Q12965 | MYO1E | 2 | 6 |
| C9J9K3 | RPSA | 5 | 6 |
| P31327 | CPS1 | 4 | 6 |
| P00966 | ASS1 | 3 | 6 |
| Q96EB6 | SIRT1 | 3 | 6 |
| P62081 | RPS7 | 4 | 6 |
| Q7L014 | DDX46 | 4 | 6 |
| Q9BU76 | MMTAG2 | 4 | 6 |
| Q8NE71 | ABCF1 | 3 | 6 |
| P12268 | IMPDH2 | 5 | 6 |
| P13639 | EEF2 | 6 | 6 |
| P53621 | COPA | 3 | 6 |
| P58107 | EPPK1 | 3 | 6 |
| Q12955 | ANK3 | 2 | 6 |
| P98161 | PKD1 | 3 | 6 |
| Q8TCU4 | ALMS1 | 3 | 5 |
| P11532 | DMD | 4 | 5 |
| P12270 | TPR | 5 | 5 |
| Q00610 | CLTC | 4 | 5 |
| P00338 | LDHA | 4 | 5 |
| P26368 | U2AF2 | 4 | 5 |
| P13533 | MYH6 | 5 | 5 |
| Q15746 | MYLK | 1 | 5 |
| P51610 | HCFC1 | 5 | 5 |
| Q6PKG0 | LARP1 | 4 | 5 |
| Q15029 | EFTUD2 | 3 | 5 |
| P46940 | IQGAP1 | 4 | 5 |
| P06748 | NPM1 | 5 | 5 |
| O60506 | SYNCRIP | 5 | 5 |
| Q07065 | CKAP4 | 4 | 5 |
| P68104 | EEF1A1 | 3 | 5 |
| O76094 | SRP72 | 5 | 5 |
| P07814 | EPRS | 5 | 5 |
| P46777 | RPL5 | 5 | 5 |
| P52597 | HNRNPF | 5 | 5 |
| P30050 | RPL12 | 5 | 5 |
| Q9HCE1 | MOV10 | 5 | 5 |
| P19105 | MYL12A | 4 | 5 |
| P29692 | EF1D | 5 | 5 |
| P62314 | SMD1 | 5 | 5 |
| Q96A72 | MGN2 | 5 | 5 |
| P53999 | TCP4 | 5 | 5 |
| P07195 | LDHB | 4 | 4 |
| P68366 | TBA4A | 4 | 4 |
| P80723 | BASP1 | 4 | 4 |
| P18085 | ARF4 | 3 | 4 |
| P22532 | SPR2D | 4 | 4 |
| P26599 | PTBP1 | 4 | 4 |
| P05198 | EIF2S1 | 2 | 4 |
| P49411 | TUFM | 3 | 4 |
| P62424 | RPL7A | 4 | 4 |
| Q9BQG0 | MYBBP1A | 4 | 4 |
| P26373 | RPL13 | 2 | 4 |
| P04844 | RPN2 | 4 | 4 |
| P25705 | ATP5A1 | 3 | 4 |
| P09651 | HNRNPA1 | 4 | 4 |
| P62857 | RPS28 | 4 | 4 |
| Q9GZR7 | DDX24 | 4 | 4 |
| Q2NL82 | TSR1 | 4 | 4 |
| P04843 | RPN1 | 3 | 4 |
| P22531 | SPR2E | 4 | 4 |
| O43707 | ACTN4 | 3 | 4 |
| Q01469 | FABP5 | 3 | 4 |
| P62241 | RPS8 | 4 | 4 |
| Q92499 | DDX1 | 4 | 4 |
| Q9UQ35 | SRRM2 | 4 | 4 |
| Q99575 | POP1 | 2 | 4 |
| P02545 | LMNA | 3 | 4 |
| P78527 | PRKDC | 4 | 4 |
| P20042 | EIF2S2 | 4 | 4 |
| Q99714 | HCD2 | 4 | 4 |
| Q96E41 | TBL2 | 3 | 4 |
| O00299 | CLIC1 | 4 | 4 |
| P52943 | CRIP2 | 4 | 4 |
| O43143 | DHX15 | 4 | 4 |
| Q00341 | HDLBP | 4 | 4 |
| Q60FE5 | FLNA | 4 | 4 |
| Q9NV31 | IMP3 | 4 | 4 |
| P62136 | PP1A | 4 | 4 |
| P61978 | HNRNPK | 3 | 4 |
| P50914 | RPL14 | 4 | 4 |
| P82650 | MRPS22 | 4 | 4 |
| Q6P1L8 | RM14 | 5 | 5 |
| Q13185 | CBX3 | 4 | 5 |
| Q9H6S0 | YTHDC2 | 4 | 5 |
| Q12906 | ILF3 | 5 | 5 |
| P62899 | RPL31 | 5 | 5 |
| Q9P258 | RCC2 | 4 | 5 |
| Q92841 | DDX17 | 5 | 5 |
| P42766 | RPL35 | 4 | 5 |
| Q8IYB3 | SRRM1 | 5 | 5 |
| Q9BXS5 | AP1M1 | 4 | 5 |
| P62888 | RPL30 | 4 | 5 |
| P53396 | ACLY | 1 | 5 |
| P78371 | TCPB | 5 | 5 |
| P51116 | FXR2 | 4 | 5 |
| P62280 | RPS11 | 5 | 5 |
| Q8NC51 | SERBP1 | 3 | 5 |
| Q96CW1 | AP2M1 | 5 | 5 |
| P51114 | FXR1 | 5 | 5 |
| P46783 | RPS10 | 4 | 5 |
| Q7L2J0 | MEPCE | 5 | 5 |
| Q2TAY7 | SMU1 | 5 | 5 |
| P19338 | NCL | 4 | 5 |
| P39019 | RPS19 | 4 | 5 |
| P62829 | RPL23 | 4 | 5 |
| Q01081 | U2AF1 | 4 | 5 |
| Q9Y383 | LUC7L2 | 3 | 5 |
| Q86V81 | ALYREF | 5 | 5 |
| P62266 | RPS23 | 4 | 5 |
| Q9H0U3 | MAGT1 | 5 | 5 |
| Q9UHX1 | PUF60 | 5 | 5 |
| Q9Y2X3 | NOP58 | 5 | 5 |
| P62277 | RPS13 | 4 | 5 |
| P14618 | PKM | 4 | 5 |
| P62249 | RPS16 | 5 | 5 |
| Q9NVP1 | DDX18 | 5 | 5 |
| P49411 | EFTU | 5 | 5 |
| P37802 | TAGL2 | 4 | 5 |
| Q9BVP2 | GNL3 | 4 | 5 |
| O00567 | NOP56 | 2 | 5 |
| Q9Y3I0 | RTCB | 4 | 5 |
| P46781 | RPS9 | 5 | 5 |
| Q14498 | RBM39 | 4 | 5 |
| O75534 | CSDE1 | 4 | 5 |
| Q13243 | SRSF5 | 3 | 5 |
| Q14690 | PDCD11 | 4 | 5 |
| Q05639 | EEF1A2 | 5 | 5 |
| Q9NUQ6 | SPATS2L | 3 | 5 |
| Q12905 | ILF2 | 5 | 5 |
| O15371 | EIF3D | 5 | 5 |
| P46779 | RPL28 | 5 | 5 |
| P15924 | DSP | 3 | 3 |
| P51398 | DAP3 | 2 | 3 |
| P78362 | SRPK2 | 1 | 3 |
| Q14331 | FRG1 | 2 | 3 |
| P06576 | ATP5B | 3 | 3 |
| Q08J23 | NSUN2 | 3 | 3 |
| P17987 | TCP1 | 3 | 3 |
| O00425 | IGF2BP3 | 2 | 3 |
| Q14151 | SAFB2 | 3 | 3 |
| P35659 | DEK | 3 | 3 |
| Q9Y5P6 | GMPPB | 3 | 3 |
| Q9P275 | UBP36 | 3 | 3 |
| O94776 | MTA2 | 3 | 3 |
| Q6WKZ4 | RFIP1 | 2 | 3 |
| Q4G0F5 | VP26B | 1 | 3 |
| P43490 | NAMPT | 3 | 3 |
| Q96IR7 | HPDL | 2 | 3 |
| Q9Y3R4 | NEUR2 | 3 | 3 |
| O15269 | SPTC1 | 3 | 3 |
| Q92621 | NU205 | 3 | 3 |
| PAXB1 | PAXB1 | 3 | 3 |
| PAXB1 | PAXB1 | 3 | 3 |
| Q9UBP6 | TRMB | 3 | 3 |
| Q96P70 | IPO9 | 3 | 3 |
| Q96BN8 | OTUL | 3 | 3 |
| Q13505 | MTX1 | 3 | 3 |
| Q86UP2 | KTN1 | 3 | 3 |
| O14929 | HAT1 | 2 | 3 |
| Q8NBX0 | SCPDL | 2 | 3 |
| P11177 | ODPB | 3 | 3 |
| P00491 | PNPH | 2 | 3 |
| Q01813 | PFKAP | 3 | 3 |
| Q15365 | PCBP1 | 3 | 3 |
| O75937 | DNJC8 | 3 | 3 |
| Q13601 | KRR1 | 3 | 3 |
| O00429 | DNM1L | 3 | 3 |
| Q9ULC4 | MCTS1 | 1 | 3 |
| P01876 | IGHA1 | 3 | 3 |
| P55036 | PSMD4 | 3 | 3 |
| Q9H089 | LSG1 | 3 | 3 |
| P0DOX2 | IGA2 | 2 | 3 |
| P60903 | S10AA | 3 | 3 |
| P41091 | IF2G | 1 | 3 |
| P26641 | EF1G | 2 | 3 |
| P52292 | KPNA2 | 1 | 3 |
| P68363 | TUBA1B | 3 | 3 |
| O00410 | IPO5 | 2 | 3 |
| Q96I24 | FUBP3 | 1 | 3 |
| Q9H299 | SH3BGRL3 | 2 | 3 |
| P62304 | SNRPE | 3 | 3 |
| P63208 | SKP1 | 3 | 3 |
| Q12874 | SF3A3 | 2 | 3 |
| P61247 | RPS3A | 3 | 3 |
| P38646 | HSPA9 | 1 | 2 |
| Q9UBT2 | UBA2 | 2 | 2 |
| P19474 | TRIM21 | 1 | 2 |
| Q9Y285 | SYFA | 2 | 2 |
| Q14692 | BMS1 | 2 | 2 |
| Q9Y446 | PKP3 | 2 | 2 |
| P22314 | UBA1 | 2 | 2 |
| Q09666 | AHNK | 2 | 2 |
| Q9BSC4 | NOL10 | 2 | 2 |
| Q9NTK5 | OLA1 | 2 | 2 |
| Q16543 | CDC37 | 1 | 2 |
| O00566 | MPP10 | 2 | 2 |
| Q14651 | PLSI | 2 | 2 |
| P62195 | PRS8 | 2 | 2 |
| Q8NAV1 | PR38A | 2 | 2 |
| Q14232 | EI2BA | 1 | 1 |
| Q8IWX8 | CHERP | 1 | 1 |
| Q9NPD8 | UBE2T | 1 | 1 |
| P00568 | KAD1 | 1 | 1 |
| P83111 | LACTB | 1 | 1 |
| Q9BRU9 | UTP23 | 1 | 1 |
| P18754 | RCC1 | 1 | 1 |
| Q15008 | PSMD6 | 1 | 1 |
| Q96J01 | THOC3 | 1 | 1 |
| Q5RKV6 | EXOS6 | 1 | 1 |
| O00483 | NDUA4 | 1 | 1 |
| Q9P2E9 | RRBP1 | 1 | 1 |
| Q08211 | DHX9 | 1 | 1 |
| P36578 | RPL4 | 1 | 1 |
| Q00839 | HNRNPU | 1 | 1 |
| P35579 | MYH9 | 1 | 1 |
| O75643 | SNRNP200 | 1 | 1 |
| P08238 | HSP90AB1 | 1 | 1 |
| P63244 | GNB2L1 | 1 | 1 |
| O60841 | EIF5B | 1 | 1 |
| Q13263 | [TRIM28](http://deer.med.harvard.edu/cgi-bin/consensus_html4?Sum=/home/www/production/html/saved_sets/34307.1.sumlist&Ref=TRIM28_IPI:IPI00438229.2&Db=/database/static/2009-07-06_FWDipi.HUMAN_gene.v.3.60.fasta&Pep=IVAERPGTNSTGPAPMAPPR+SGEGEVSGLMR+SGEGEVSGLMR) | 1 | 1 |
| Q92618 | [ZNF516](http://deer.med.harvard.edu/cgi-bin/consensus_html4?Sum=/home/www/production/html/saved_sets/34307.1.sumlist&Ref=ZNF516_IPI:IPI00852669.1&Db=/database/static/2009-07-06_FWDipi.HUMAN_gene.v.3.60.fasta&Pep=ALAPDLMPLDLSAR+SGSSPLGVVTK+HSAPDSLK) | 1 | 1 |
| Q86UE4 | [MTDH](http://deer.med.harvard.edu/cgi-bin/consensus_html4?Sum=/home/www/production/html/saved_sets/34307.1.sumlist&Ref=MTDH_IPI:IPI00328715.4&Db=/database/static/2009-07-06_FWDipi.HUMAN_gene.v.3.60.fasta&Pep=SWQDELAQQAEEGSAR+LSSQISAGEEK) | 1 | 1 |

PSMs: peptide-spectrum matches
