# Supplementary material for: Nuclear isoform of RAPH1 interacts with FOXQ1 to promote aggressiveness and radioresistance in breast cancer
Source: Cell Death Dis. 2023 Dec 7;14(12):803. doi: 10.1038/s41419-023-06331-9 (PMC10703867; doi:10.1038/s41419-023-06331-9)

**Original data files**

Figure 1B


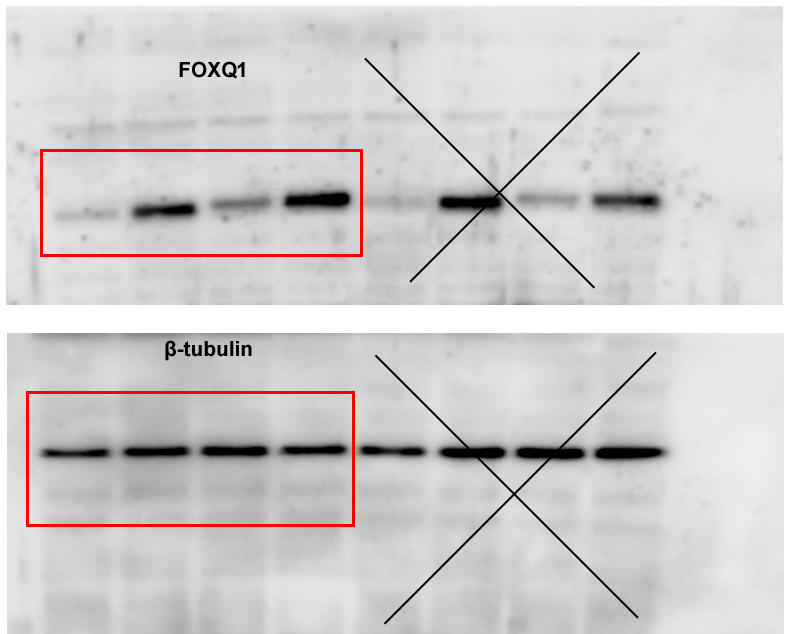


Figure 2C


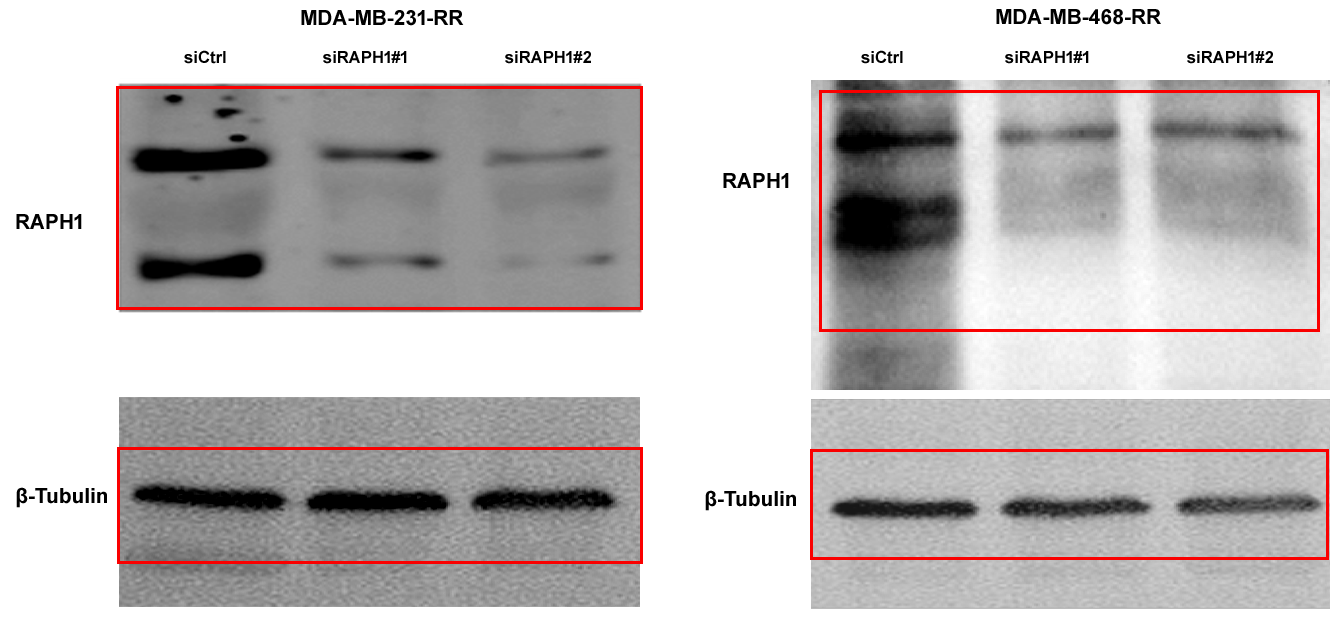


Figure 3A


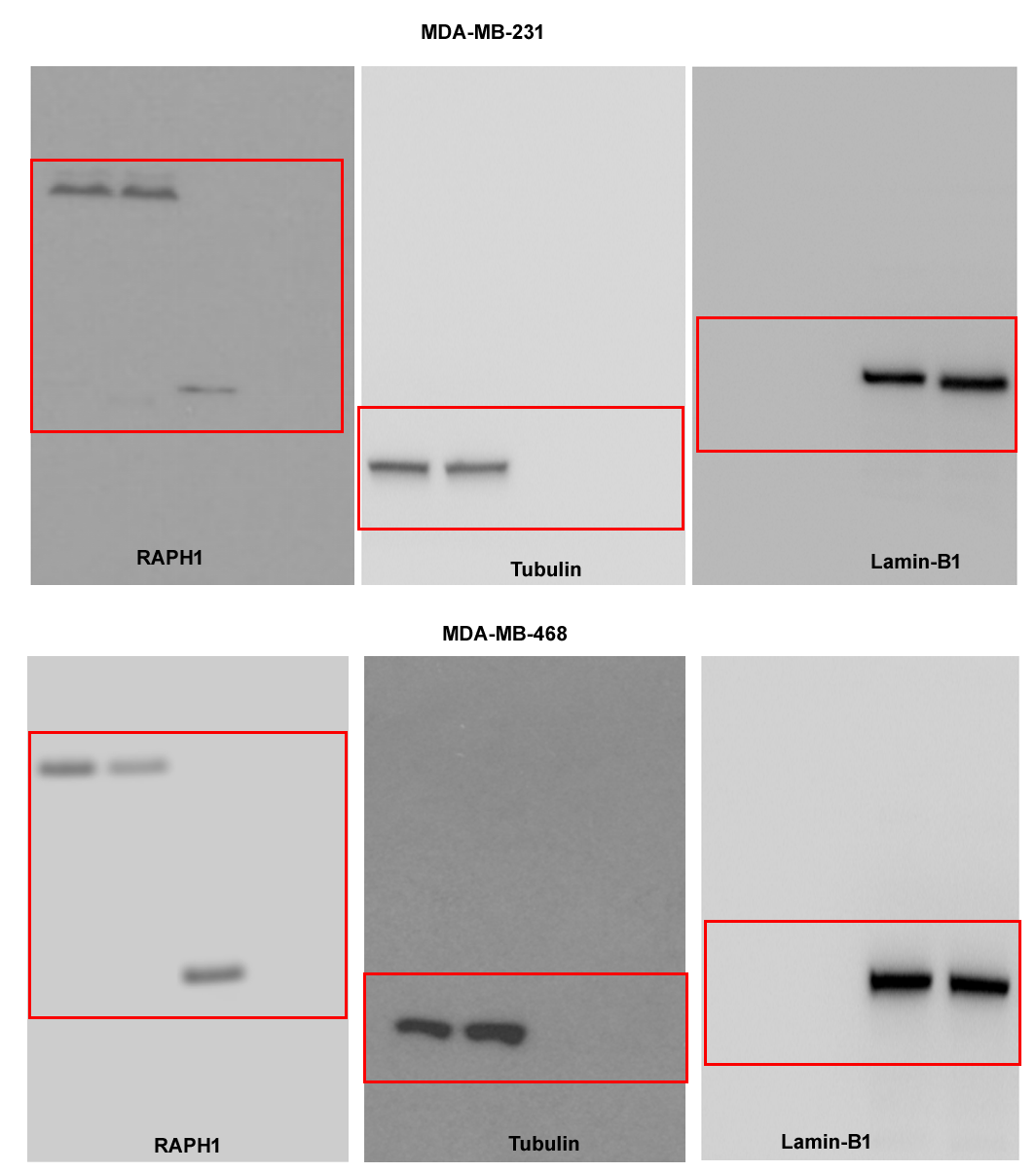


Figure 3E


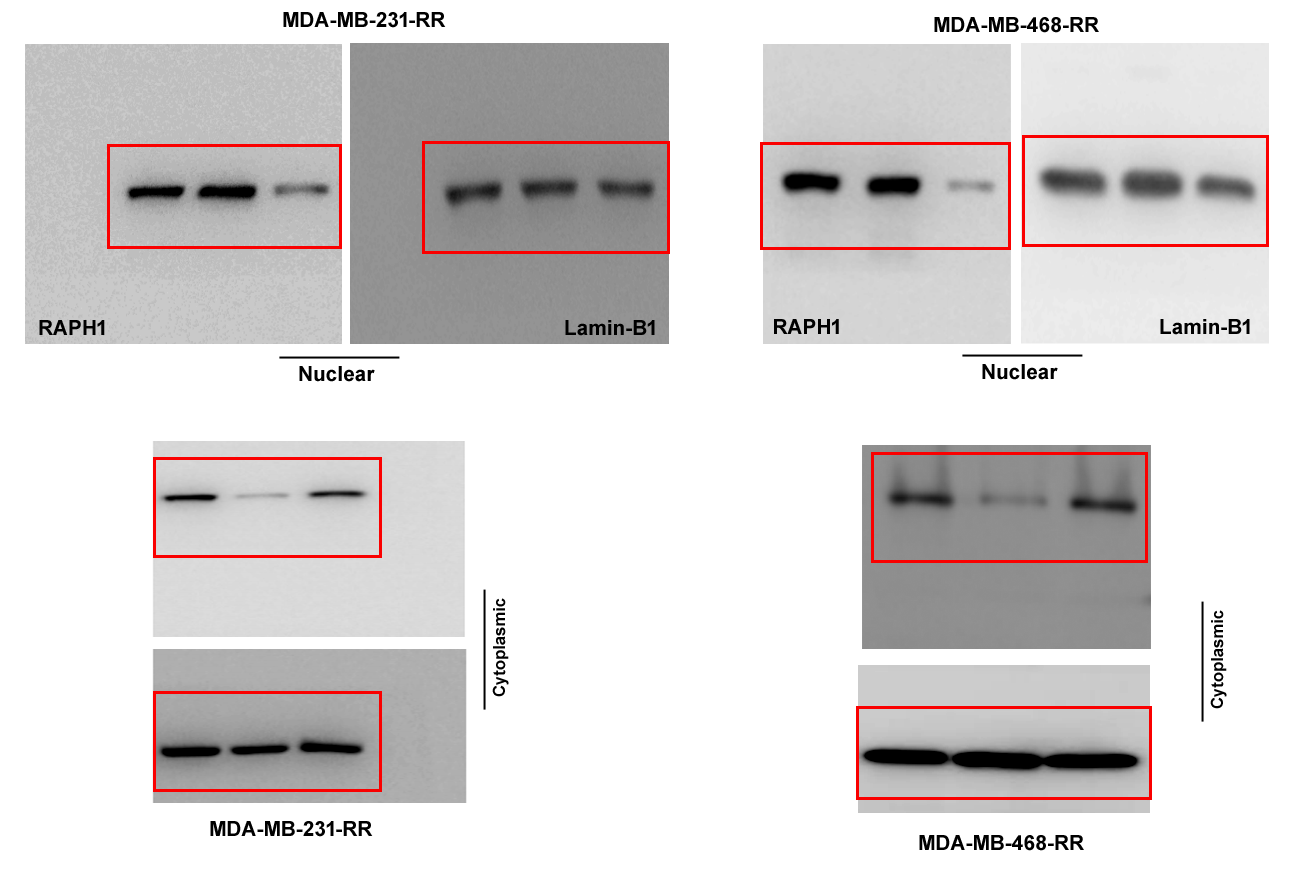


Figure 4A


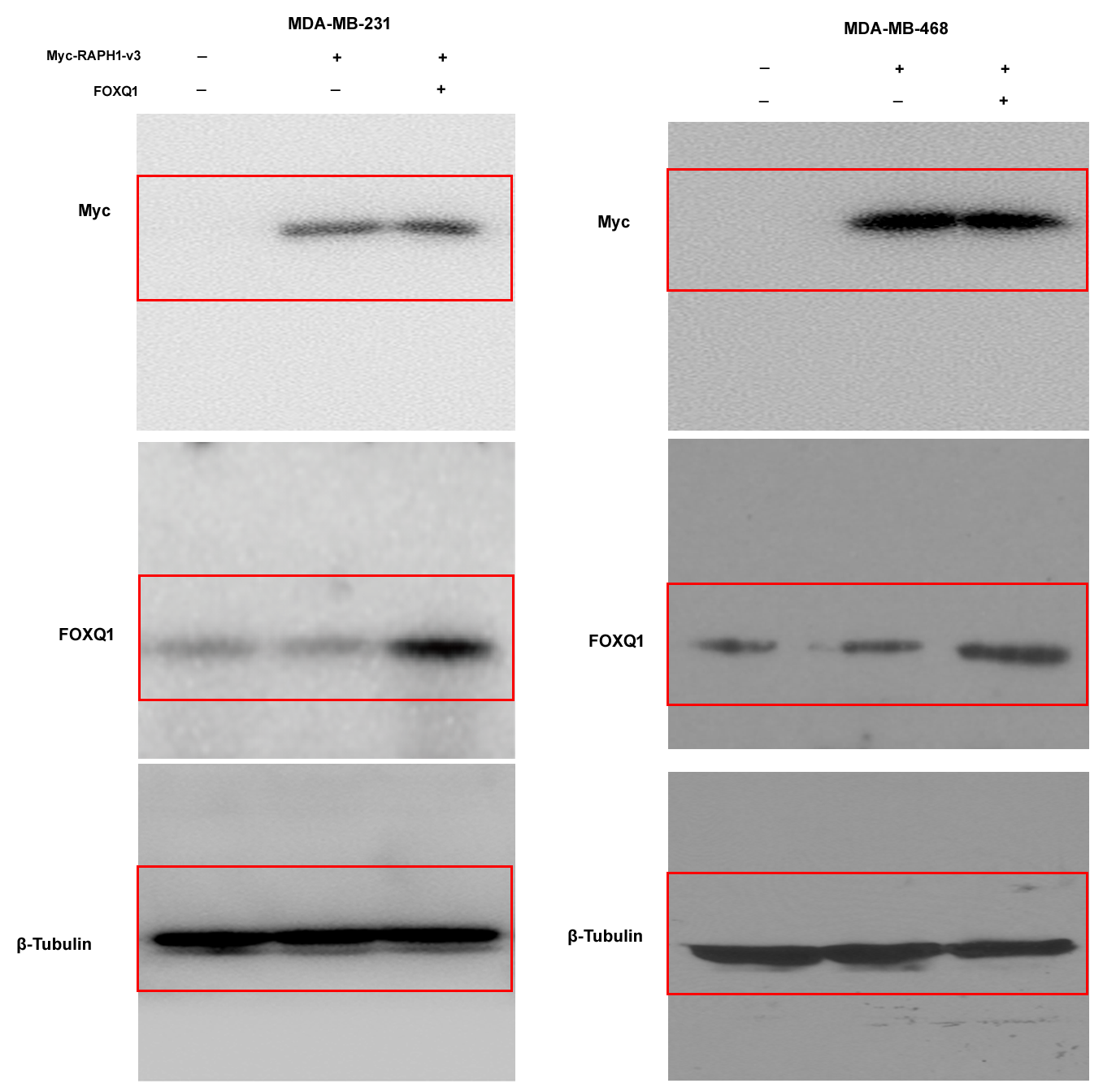


Figure 5A


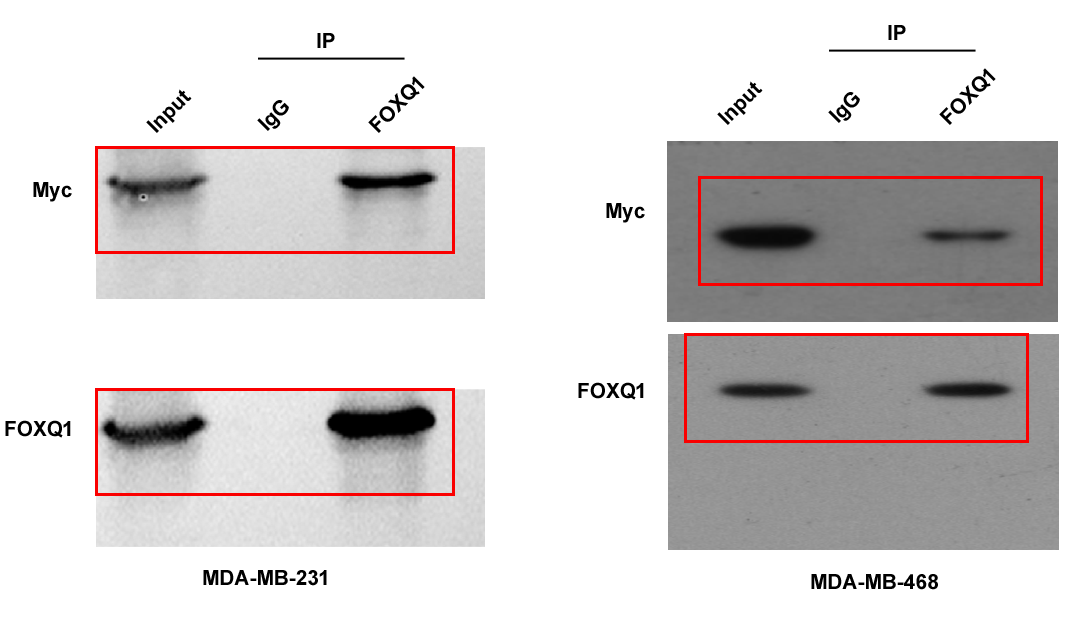


Figure 5E


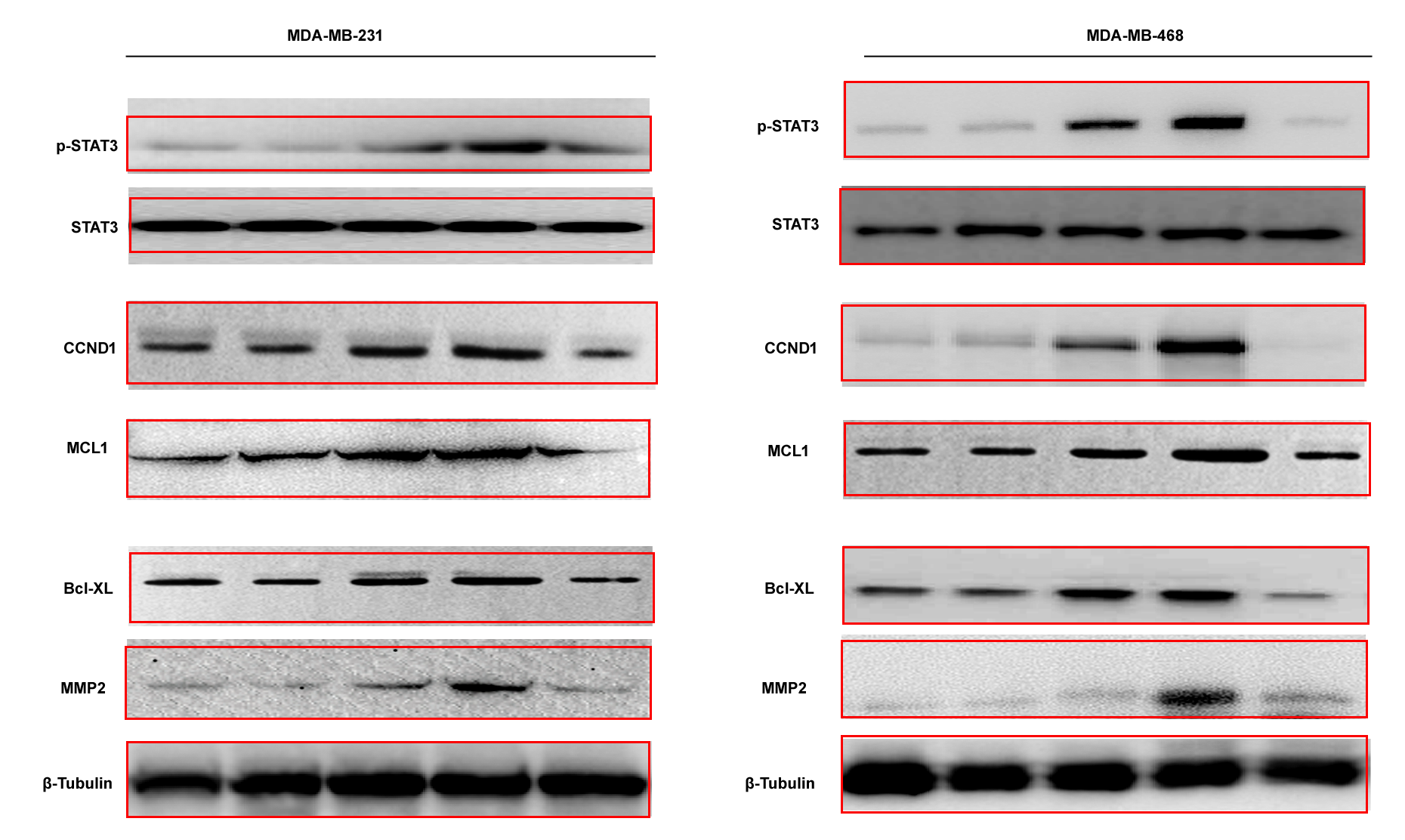


Figure 6G, 6J


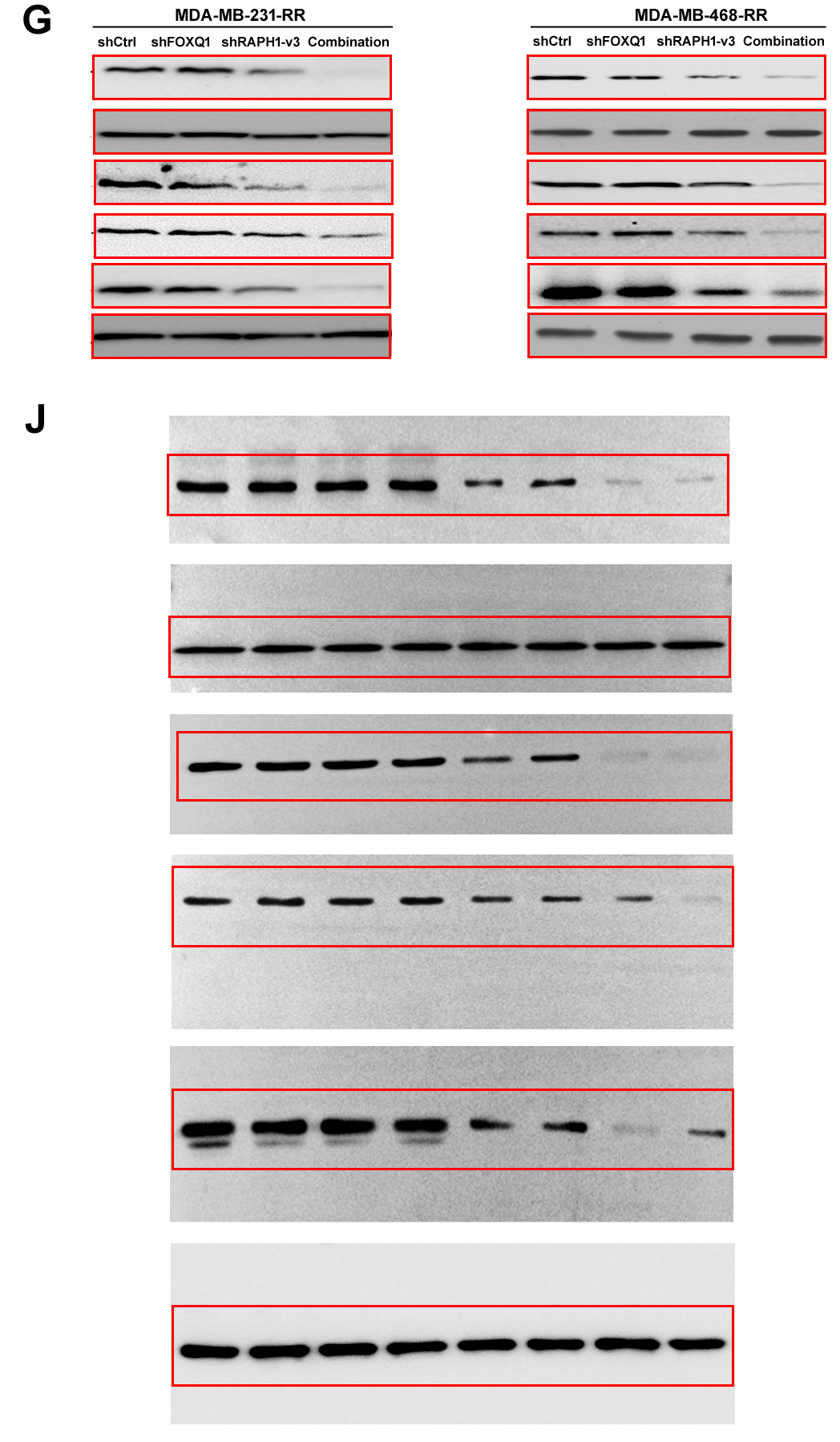


Figure S1


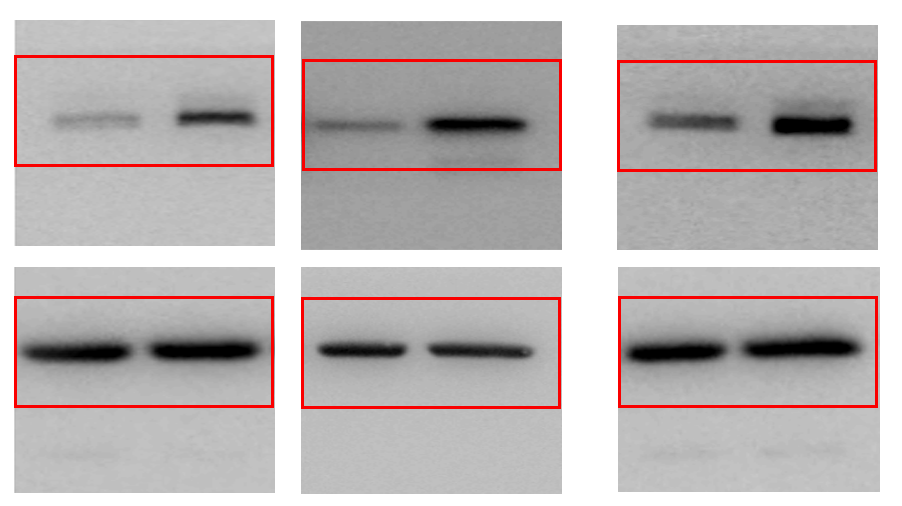


Figure S2


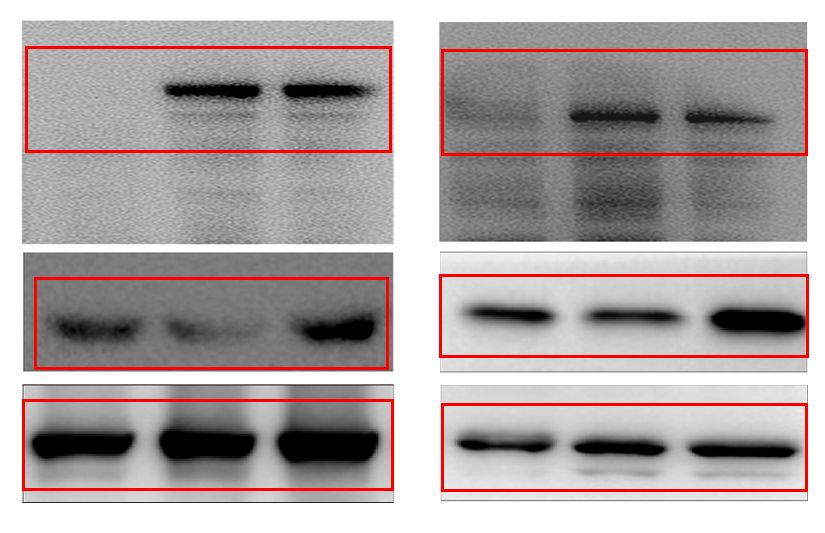


Figure S3


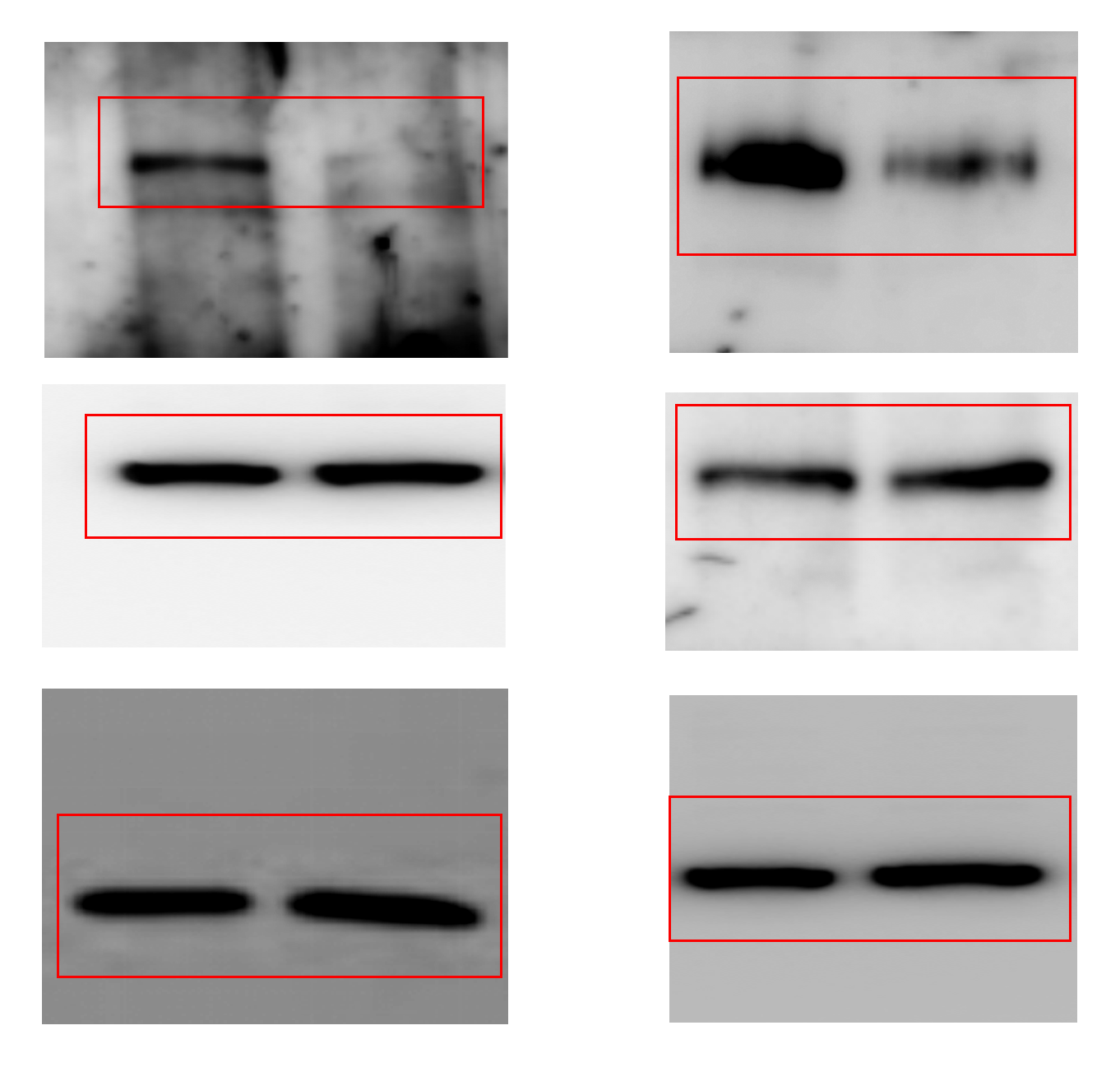


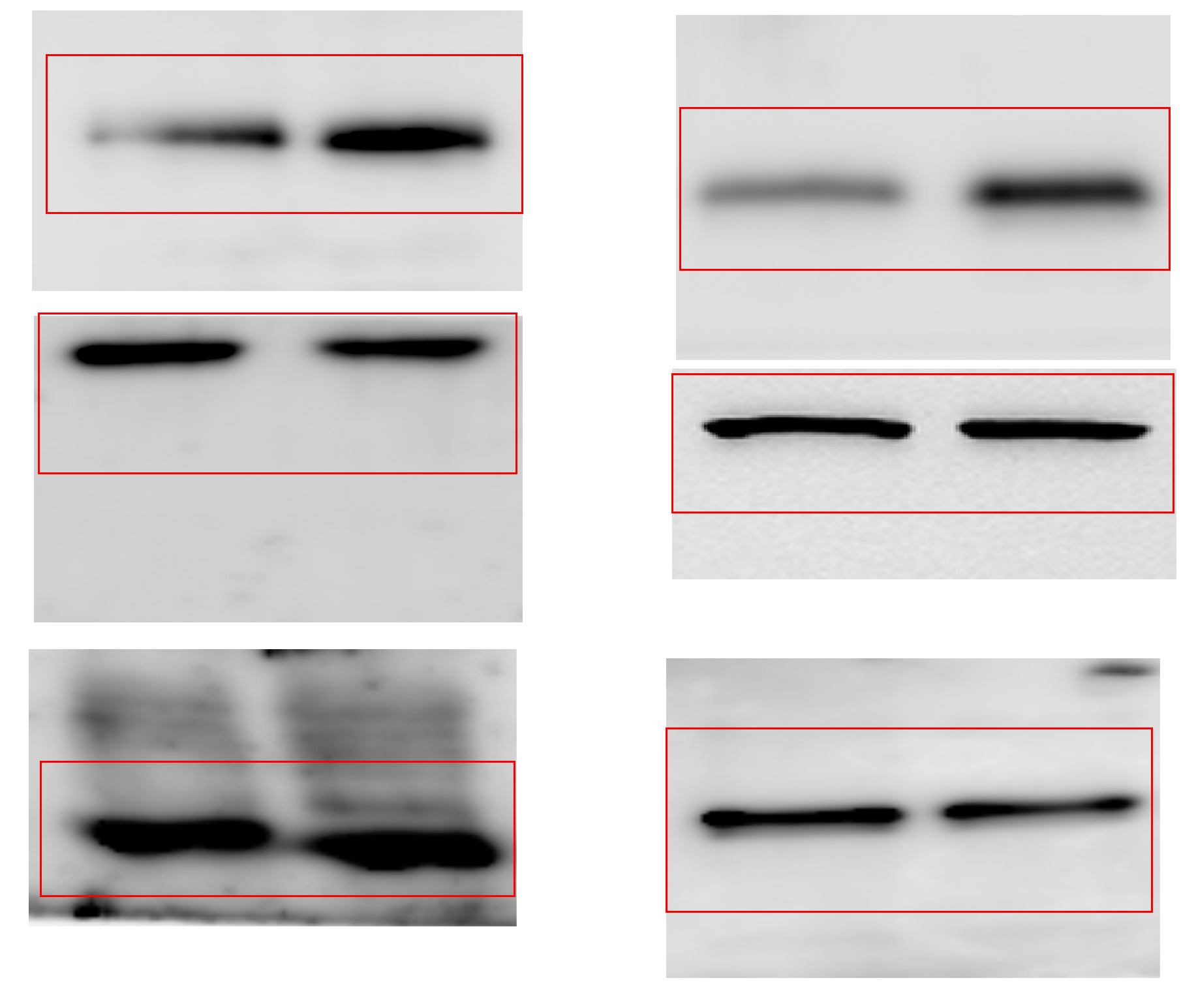


Figure S4


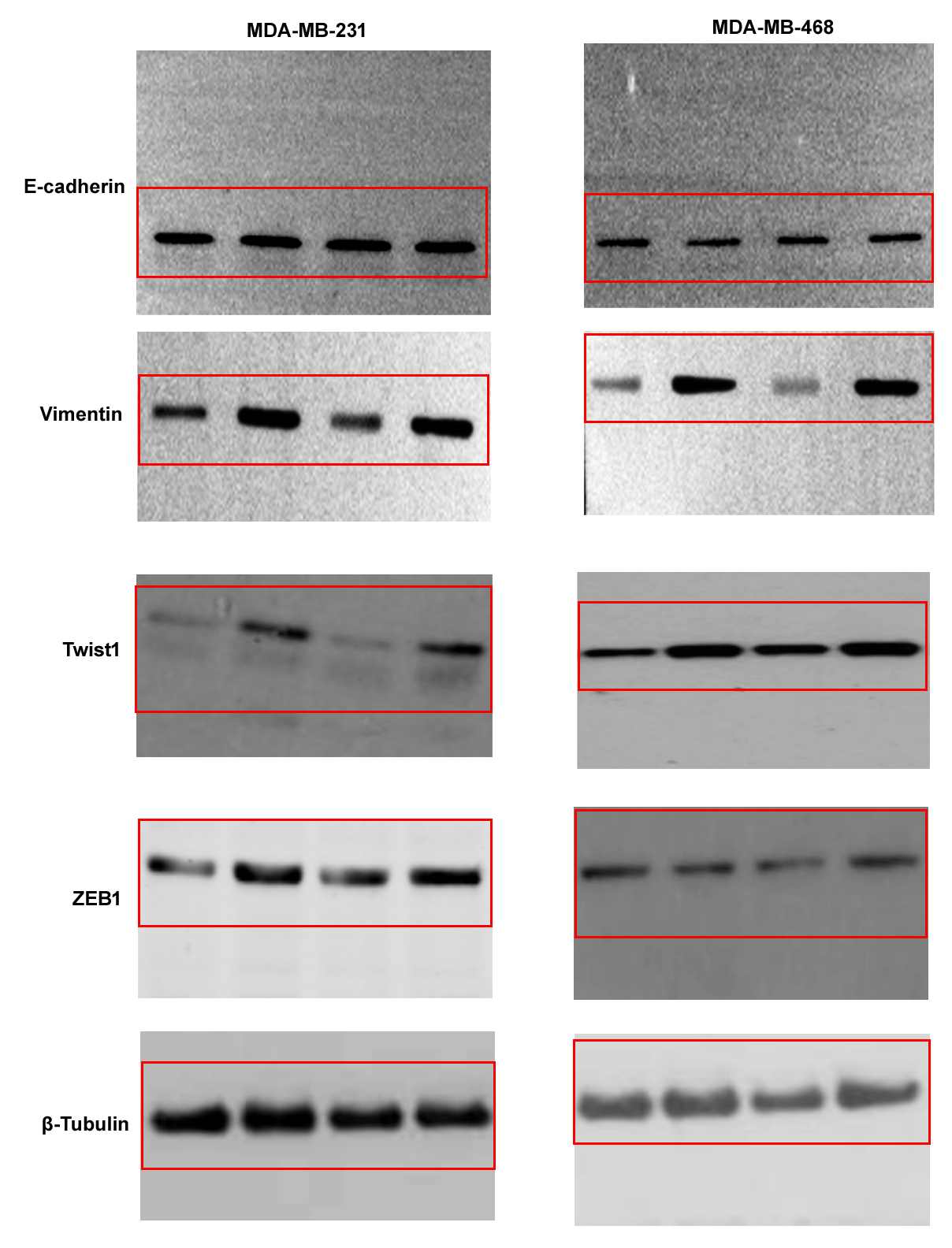


Figure S5


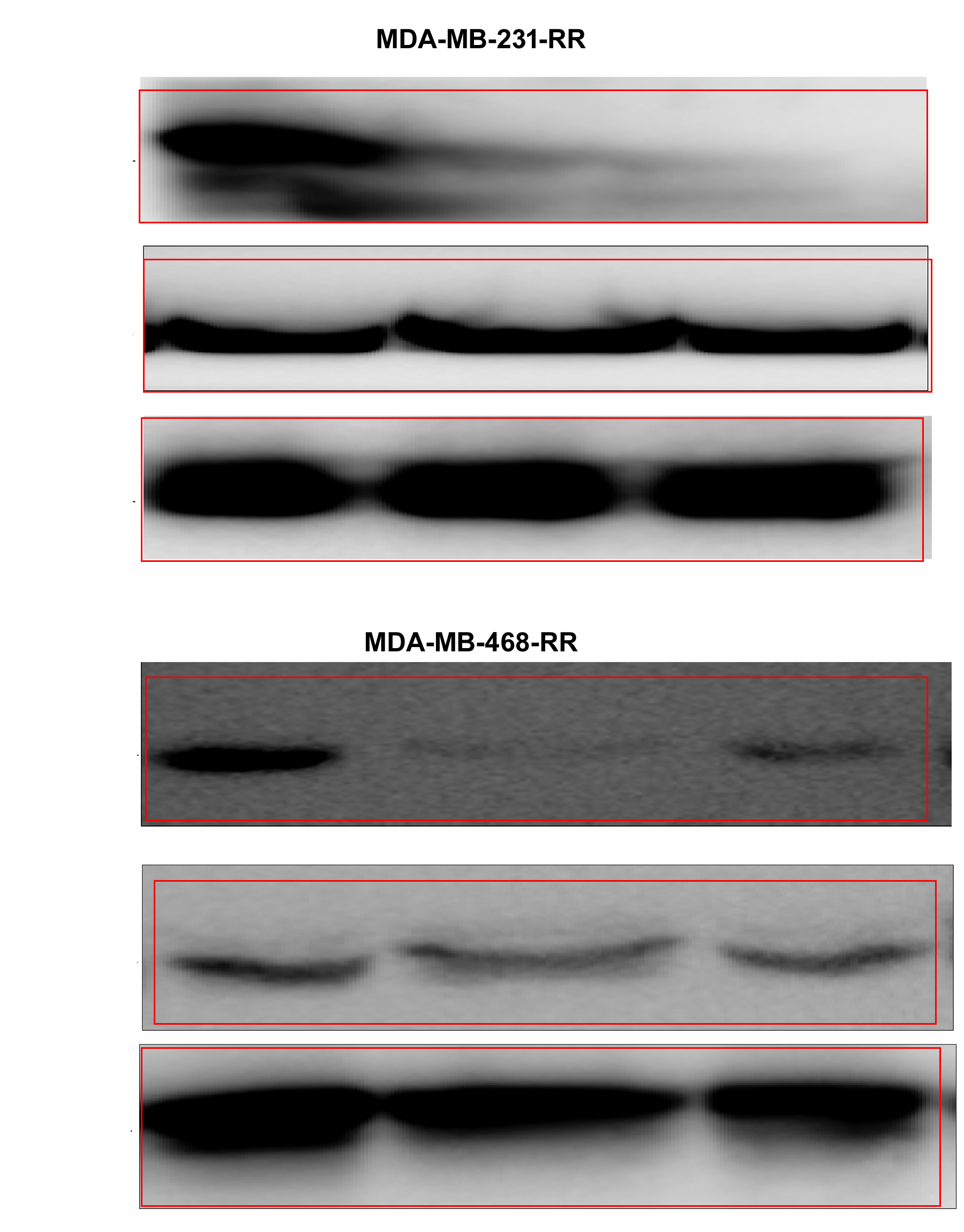

Supplement: Supplementary file 2 — Original Data File [file 41419_2023_6331_MOESM2_ESM.doc]
